# Preclinical evaluation of eltrombopag in a PDX model of myelodysplastic syndromes

**DOI:** 10.1038/s41375-021-01327-w

**Published:** 2021-06-25

**Authors:** Nanni Schmitt, Johann-Christoph Jann, Eva Altrock, Johanna Flach, Justine Danner, Stefanie Uhlig, Alexander Streuer, Antje Knaflic, Vladimir Riabov, Qingyu Xu, Arwin Mehralivand, Iris Palme, Verena Nowak, Julia Obländer, Nadine Weimer, Verena Haselmann, Ahmed Jawhar, Ali Darwich, Cleo-Aron Weis, Alexander Marx, Laurenz Steiner, Mohamad Jawhar, Georgia Metzgeroth, Tobias Boch, Florian Nolte, Wolf-Karsten Hofmann, Daniel Nowak

**Affiliations:** 1 Department of Hematology and Oncology, Medical Faculty Mannheim, Heidelberg University, Mannheim, Germany; 2 Flow Core Mannheim and Institute of Transfusion Medicine and Immunology, Medical Faculty Mannheim, Heidelberg University, Mannheim, Germany; 3 Institute of Clinical Chemistry, Medical Faculty Mannheim, Heidelberg University, Mannheim, Germany; 4 Department of Orthopedics and Traumatology, Medical Faculty Mannheim, Heidelberg University, Mannheim, Germany; 5 Institute of Pathology, Medical Faculty Mannheim, Heidelberg University, Mannheim, Germany

**Keywords:** Cancer models, Preclinical research, Myelodysplastic syndrome

## Abstract

Preclinical research of myelodysplastic syndromes (MDSs) is hampered by a lack of feasible disease models. Previously, we have established a robust patient-derived xenograft (PDX) model for MDS. Here we demonstrate for the first time that this model is applicable as a preclinical platform to address pending clinical questions by interrogating the efficacy and safety of the thrombopoietin receptor agonist eltrombopag. Our preclinical study included *n* = 49 xenografts generated from *n* = 9 MDS patient samples. Substance efficacy was evidenced by FACS-based human platelet quantification and clonal bone marrow evolution was reconstructed by serial whole-exome sequencing of the PDX samples. In contrast to clinical trials in humans, this experimental setup allowed vehicle- and replicate-controlled analyses on a patient–individual level deciphering substance-specific effects from natural disease progression. We found that eltrombopag effectively stimulated thrombopoiesis in MDS PDX without adversely affecting the patients’ clonal composition. In conclusion, our MDS PDX model is a useful tool for testing new therapeutic concepts in MDS preceding clinical trials.

## Introduction

Myelodysplastic syndromes (MDSs) are clonal hematopoietic stem cell disorders characterized by bone marrow (BM) dysplasia, cytopenias, recurrent genomic aberrations, and a predisposition for progression to acute myeloid leukemia [1]. Although there has been great progress in the molecular characterization of MDS [2], the translation of this mutational data into novel treatment strategies is frequently impeded by a lack of preclinical models to address functional questions. To overcome these limitations, we have previously established a niche-based patient-derived xenograft (PDX) model in NOD.Cg-*Prkdc*^scid^ *Il2rg*^tm1Wjl^/SzJ (NSG) mice consisting of an orthotopic co-transplantation of ex vivo expanded BM-derived mesenchymal stroma cells (MSCs) and CD34+ hematopoietic stem cells [3]. Although other MDS PDX models have been demonstrated as proof of principle [4, 5], in our current study we aimed at demonstrating that our established MDS PDX model can indeed serve as a preclinical platform to address clinical questions. To this end, we here investigated thrombocytopenia in MDS patients, which is a frequent condition accountable for life-threatening hemorrhagic events and represents an independent adverse prognostic factor [6]. Its estimated prevalence in MDS patients ranges from 40% to 65% [7]. Moreover, current treatment options for MDS such as lenalidomide or 5-azacytidine may worsen pre-existing thrombocytopenias, ultimately requiring dose reduction or interruption of treatment [8–10]. Therefore, many MDS patients are dependent on supportive platelet (PLT) transfusions, which in turn are associated with considerable risks [7, 11].

In this context, thrombopoietin (Tpo) receptor agonists such as eltrombopag (EPAG) have shown efficacy in clinical trials as alternative treatment options for thrombocytopenic MDS patients [12–15]. However, concerns about a potential promotion of disease progression in MDS by these substances based on the Tpo receptor’s ubiquitous expression on hematopoietic stem cells [16–18] have not entirely been ruled out. Due to the high clinical and molecular heterogeneity among MDS patients, these trials faced the fundamental limitation of not being able to distinguish potential substance-induced clonal evolution from natural disease progression in the same patient. To address this issue, preclinical modeling of MDS using PDX models is the tool of choice, as they allow the generation of multiple replicates and therefore vehicle-controlled experiments on a patient–individual level.

## Materials and methods

### Patient samples

MDS patient samples were obtained from diagnostic BM aspirations (BMAs) after informed written consent and in accordance with the Declaration of Helsinki. Mononuclear cells (MNCs) were isolated using Ficoll-Paque (Cytiva, Freiburg, Germany) density gradient centrifugation. CD34+ cell enrichment from MNCs was performed using MACS columns (Miltenyi Biotec, Bergisch Gladbach, Germany). Patient MSCs were selected by plastic adherence and expanded ex vivo with StemMACS MSC Expansion Media XF (Miltenyi Biotec).

### Mouse experiments

Xenotransplantation experiments were performed using a modified protocol according to Medyouf et al. [3]. In brief, 8-week-old female NSG mice (Jackson Laboratory, Bar Harbor, ME, USA) were sublethally conditioned with Busulfan (25 mg/kg) intraperitoneally 48 and 24 h prior to bilateral intrafemoral injection of CD34+ cells and autologous MSCs. Long-term engraftment was assessed 12 weeks post transplant. Mice with positive human engraftment received oral treatment of either EPAG (50–150 mg/kg) or vehicle for 18–24 weeks until endpoint. During the treatment period, cheek bleeding was performed biweekly and BMA on the same leg every 6 weeks. At endpoint, the bones (tibia, femur, pelvis, humerus, spine) were crushed to extract BM. Animal experiments were approved by the state authority Karlsruhe, Germany.

### Flow cytometry

BM from PDX was blocked with human (h) and mouse (m) FcR Blocking Reagent (Miltenyi Biotec), and stained with the antibodies listed in Supplementary Table S1. Dead cells were excluded using SYTOX Blue (Thermo Fisher Scientific, Waltham, MA, USA). Analysis and sorting of hCD45+ and hCD45+ hCD61+ cells were performed using a BD FACSMelody and BD FACSAria II cell sorter (Becton Dickinson, Franklin Lakes, NJ, USA). Lysed peripheral blood (PB) from PDX was stained with hCD41 and mCD41, and analyzed using a BD FACSCanto II flow cytometer. For absolute quantification of human PLTs, Flow-Count Fluorospheres (Beckman Coulter, Brea, CA, USA) were used as described previously [19].

### Histological analysis

Detailed protocols of the performed stainings are provided in the Supplementary Methods.

### Whole-exome sequencing and bioinformatic analysis

DNA was isolated using the AllPrep DNA/RNA Kit (Qiagen, Hilden, Germany). For whole-exome sequencing (WES), 250 ng of genomic DNA from patient and xenograft samples was subjected to the Nextera DNA Flex Kit (Illumina, San Diego, CA, USA) and subsequent hybrid capture using the xGen Exome Research Panel (IDT, Coralville, IA, USA). The final library pools were sequenced on a S4 Nova Seq Flow Cell (Illumina) with 150 bp paired end. For mutational calling, raw sequencing data were subjected to a previously established bioinformatical pipeline [20] adapted for the analysis of xenograft samples. DNA from matched MSCs served as germline controls (CTRLs). On average, a mean coverage of 89.8-fold was achieved. For tracking of clonal evolution, individual clustering of variant allele frequencies (VAFs) for consecutive samples of each PDX and the corresponding transplanted patient sample was performed using the R package SciClone (v1.1) [21]. Details are provided in the Supplementary Methods.

### Sanger sequencing

DNA of hCD45+ and hCD45+ hCD61+ cells was PCR-amplified using the FastStart™ High Fidelity PCR-System (Roche, Basel, Switzerland) and a Mastercycler X50s (Eppendorf, Hamburf, Germany). Primer sequences are listed in Supplementary Table S2. PCR products were purified using the QIAquick PCR Purification Kit (Qiagen) and sequenced at Eurofins Genomics (Ebersberg, Germany).

### Statistical analysis

Data were analyzed using Prism 8 (GraphPad Software, San Diego, CA, USA). *P*-values < 0.05 were considered significant (ns, not significant; *p < 0.05; **p < 0.01; ***p < 0.001; ****p < 0.0001).

## Results

### Robust long-term engraftment in an MDS PDX model enables comprehensive preclinical substance testing and molecular readouts in vivo

**Fig. 1:**
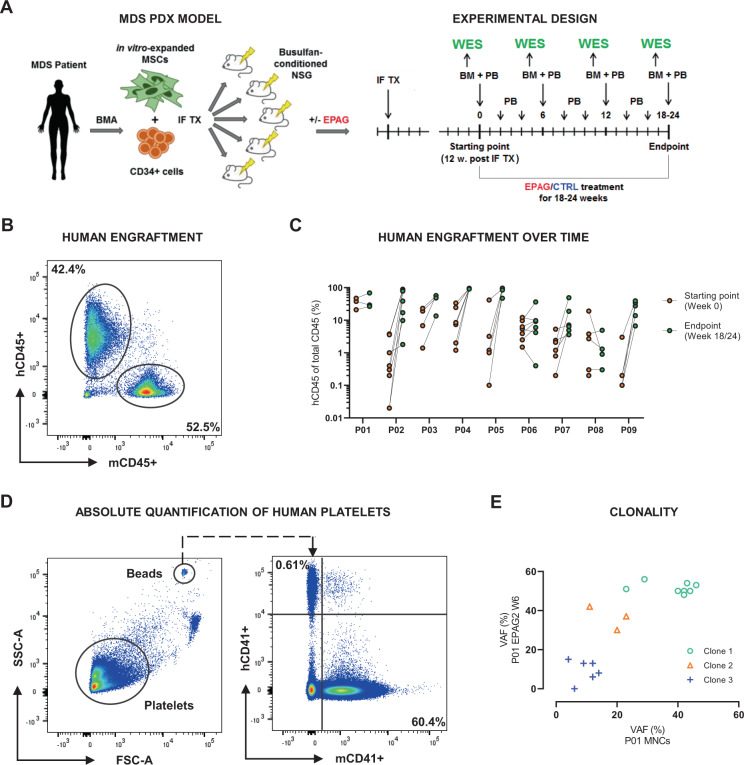
Experimental setup and readouts for preclinical assessment of EPAG using a MDS patient-derived xenograft model. **A** Schematic experimental setup. CD34+ cells and mesenchymal stromal cells (MSCs) derived from bone marrow aspirations (BMAs) of MDS patients were injected into Busulfan-conditioned NSG mice by bilateral intrafemoral transplantation (IF TX). Long-term engraftment was determined 12 weeks post transplant. Mice with positive human engraftment subsequently received oral treatment of eltrombopag (EPAG) or vehicle control (CTRL) for 18–24 weeks until endpoint. Starting with treatment, peripheral blood (PB) and BM were sampled every 2 and 6 weeks, respectively. **B** Representative flow cytometry plot showing percentage of human CD45+ (hCD45+) and mouse CD45+ (mCD45+) cells in the BM of patient-derived xenografts (PDXs). Human engraftment was defined as percentage of hCD45+ of total CD45+ cells. **C** Comparison of engraftment rates between patients’ PDX at starting (orange) and endpoint (green). On the *x*-axis, patient IDs are shown. **D** Representative flow cytometry plots showing gating scheme for platelets (PLTs) and beads (left), and percentage of hCD41+ and mCD41+ PLTs in PB of PDX (right). The absolute number of human PLTs per microliter PB was calculated in relation to the number of beads recorded. **E** Exemplary clustering of variant allele frequencies (VAFs) of mutations and copy number variations into different subclones using the bioinformatic tool SciClone. FSC-A, forward scatter area; SSC-A, side scatter area.

In the current study, we performed an evaluation of whether our previously established MDS PDX model could be used as a preclinical platform to perform MDS patient–individual substance testing. For that purpose, we aimed at evaluating the efficacy of EPAG on primary MDS cells in vivo and its influence on the clonal composition as compared to non-treatment CTRLs. We included samples of *n* = 9 MDS patients specified in Table 1. Multiple xenograft replicates of each MDS patient were generated (Fig. 1A), resulting in a cohort of *n* = 49 PDX. Twelve weeks after transplantation, xenografts were analyzed for human chimerism, defined as percentage of hCD45+ of total CD45+ cells in BMAs (Fig. 1A, B). Mice with positive human engraftment were divided into groups of EPAG and vehicle treatment (CTRL). From the start of treatment, xenografts were BM aspirated every 6 weeks, to track the percentage of human engraftment, and sort hCD45+ cells for DNA isolation and subsequent WES (Fig. 1A). Sorted hCD45+ almost exclusively consisted of myeloid lineage cells (>90% hCD45+hCD33+) (Supplementary Fig. S1). In the majority of cases, engraftment increased throughout the 18–24 weeks of experiment (Fig. 1C). Importantly, apart from single exceptions, even engraftment rates of over 90% did not affect the mice’s general health, e.g., mice generally did not develop anemia (Supplementary Fig. S2). To assess the efficacy of EPAG in PDX, PB was sampled biweekly. To quantify human PLTs in the PDX model, we measured hCD41+ PLTs and normalized them to counting beads for absolute quantification by flow cytometry (Fig. 1D). Using this approach, we could reliably monitor human PLT courses throughout the experiment. To evaluate EPAG’s influence on the PDX’s BM clonality, WES of the transplanted patient samples and all collected xenograft BM samples was performed. VAFs of mutations, copy number variations, and loss of heterozygosity events were used to infer clonal clusters using the bioinformatic tool SciClone, to reconstruct the clonal BM composition and its evolution in the course of treatment (Fig. 1E).

**Table 1:** Detailed characteristics of xenografted MDS patients.

Patient	Age (years)	Sex	WHO 2016	IPSS-R	Karyotype	Mutations	Blasts in BM (%)	PLT number (×10^E9^/L)	Prior treatment
P01	59	M	MDS-EB-2	Very high	Complex aberrant	CBL, DNMT3A, EZH2	12	29	AZA, PT, RCT
P02	82	M	MDS-EB-2	Very high	Complex aberrant	TET2, TP53	13	7	AZA, PT, RCT
P03	65	M	tMDS-EB-1	Very high	Complex aberrant	BCOR, KRAS	5	15	AZA, PT, RCT
P04	71	M	tMDS-MLD	Low	46,XY,r(6) (p21q26) [14]/46,XY [6]	ASXL1, RUNX1, SRSF2, TET2	1	24	RCT
P05	69	M	MDS-EB-1	Very high	Complex aberrant	TP53	7	15	AZA, PT, RCT
P06	82	M	MDS-EB-2	Very high	47,XY,+8 [4]/46,XY [21]	ASXL1, RUNX1, STAG2, ZRSR2	10	15	None
P07	83	F	MDS-MLD	int	46,XX	ASXL1, BCOR, CBL, DNMT3A, EZH2, TET2, WT1	4	24	G-CSF
P08	80	M	MDS-U	int	47,XY,+8 [3]/46,XY [17]	ASXL1, IDH2, SRSF2, STAG2	4	80	ESA
P09	77	M	MDS with isolated del(5q)	Very low	Del5q	ASXL1, ETV6, EZH2	1	74	Deferoxamine, Lenalidomide, PT, RCT

*AZA* 5-Azacitidine, *BM* bone marrow, *EB* with excess blasts, *ESA* erythropoiesis-stimulating agent, *G-CSF* granulocyte colony-stimulating factor, *int* intermediate, *IPSS-R* International Prognostic Scoring System–revised, *MLD* multilineage dysplasia, *PLT* platelet, *PT* platelet transfusion, *RCT* red cell transfusion, *U* unclassifiable.

**Fig. 2:**
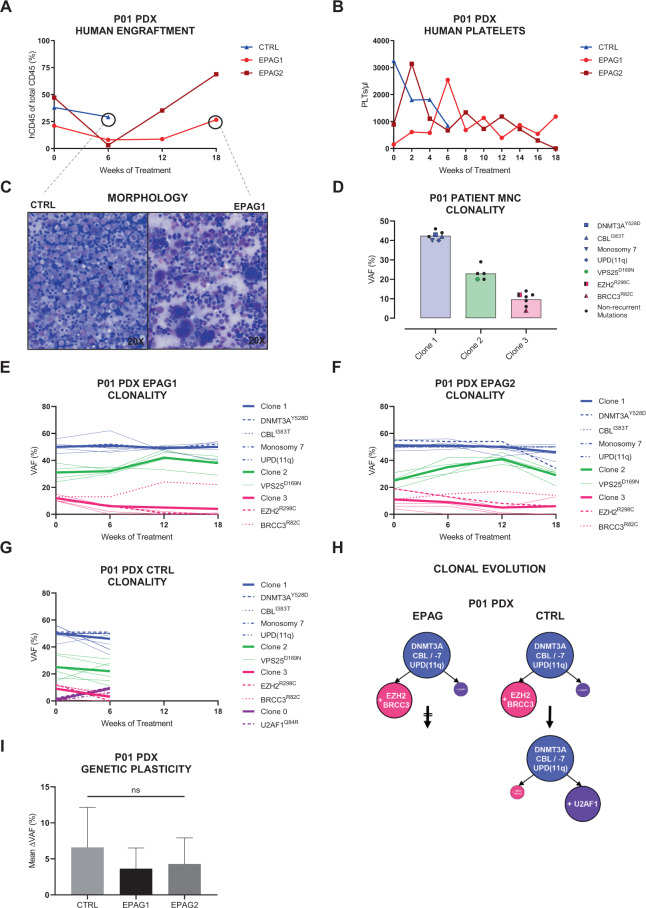
Exemplary complete overview of comprehensive clinical and molecular readouts of patient P01 and its xenografts. Of *n* = 3 xenografts, *n* = 2 were treated with eltrombopag (EPAG1 + 2, red) and *n* = 1 received vehicle control (CTRL, blue). CTRL mouse had to be killed 6 weeks into treatment phase due to excessive weight loss (see Supplementary Fig. S3). **A** Percentage of human engraftment in the bone marrow (BM) of xenografts throughout 18 weeks of treatment. Engraftment was assessed every 6 weeks. **B** Course of human platelets (PLTs) in the peripheral blood of xenografts during treatment phase. PLTs were analyzed every 2 weeks. **C** BM smears of CTRL (left) and EPAG1 (right) at endpoint stained with May–Grünwald–Giemsa stain (magnification ×20). **D** Mutational variant allele frequencies (VAFs) of primary mononuclear cell (MNC) sample of patient P01. Each bar represents one individual clone. Non-synonymous mutations are displayed with their superscripted respective amino acid change. **E**–**G** VAFs of patient-specific mutations in the course of treatment detected in the BM of EPAG1, EPAG2, and CTRL, respectively. Mutations were separated into different subclones using the bioinformatic tool SciClone. MDS-associated molecular lesions are highlighted. Thicker lines represent the mean value of the respective clone. **H** Reconstruction of differential clonal evolution in the xenografts of P01 for both treatment groups. **I** Mean deltaVAF of EPAG1, EPAG2, and CTRL determined from all identified somatic mutations for any two consecutive WES time points. Data were analyzed using one-way ANOVA and are represented as mean ± SD. ns, not significant.

Figure 2 shows an exemplary complete overview of comprehensive clinical and molecular readouts obtained from *n* = 3 PDX of patient P01. All xenografts showed high engraftment rates until their respective endpoints (Fig. 2A). The CTRL mouse had to be killed after 6 weeks of vehicle treatment due to excessive weight loss (Supplementary Fig. S3). During that time, the number of human PLTs in CTRL continuously decreased (Fig. 2B). In contrast, EPAG treatment resulted in a rise of PLT counts after 2 weeks in both mice. Although PLT numbers of EPAG2 peaked at 2 weeks and subsequently returned to baseline, EPAG1 showed a steady increase in human PLTs over 6 weeks and maintained an elevated PLT level compared to baseline until endpoint. To investigate the early deterioration of health in the untreated CTRL mouse, we assessed its BM smears for signs of transformation (Fig. 2C). Although blasts were absent, CTRL’s BM was clearly hypercellular with a monomorphic population of highly dysplastic myelocytes, whereas the EPAG-treated PDX presented with an abundance of megakaryocytes and a higher proportion of differentiating hematopoietic cells of erythro- and granulopoiesis.

To determine whether EPAG treatment affected the clonal BM composition, we performed WES on patient P01’s MNCs and all collected PDX samples. In the P01’s primary patient sample, three subclones were detected (Fig. 2D), which were faithfully recapitulated in all *n* = 3 xenografts (Fig. 2E–G). During the 18 weeks of treatment, the clonal composition in both EPAG1 and EPAG2 remained largely stable (Fig. 2E, F). In contrast to the EPAG-treated mice, a novel subclone with a *U2AF1*^Y528D^ mutation expanded in the CTRL mouse (Fig. 2G). Within 6 weeks, this clone expanded and superseded the previously present *EZH2*^R298C^/*BRCC3*^R82C^ subclone. Interestingly, the *U2AF1*^Y528D^ clone was also detectable in EPAG1 and EPAG2 but remained stable with a VAF of below 1%. Consequently, the propagation of this *U2AF1*^Y528D^ clone only occurred in the CTRL mouse but not in the EPAG-treated mice (Fig. 2H). To further visualize the degree of clonal dynamics in dependency of treatment, we calculated the ratio of total changes of VAFs over time from all identified somatic mutations for any two consecutive WES time points (mean deltaVAF) as previously described for primary MDS patient samples [21]. Although not statistically significant, the mean deltaVAF of the CTRL mouse was higher as compared to both EPAG mice, possibly indicating a higher rate of clonal evolution in the untreated condition (Fig. 2I).

### EPAG efficaciously stimulates thrombo- and megakaryopoiesis in a functional MDS PDX model

**Fig. 3:**
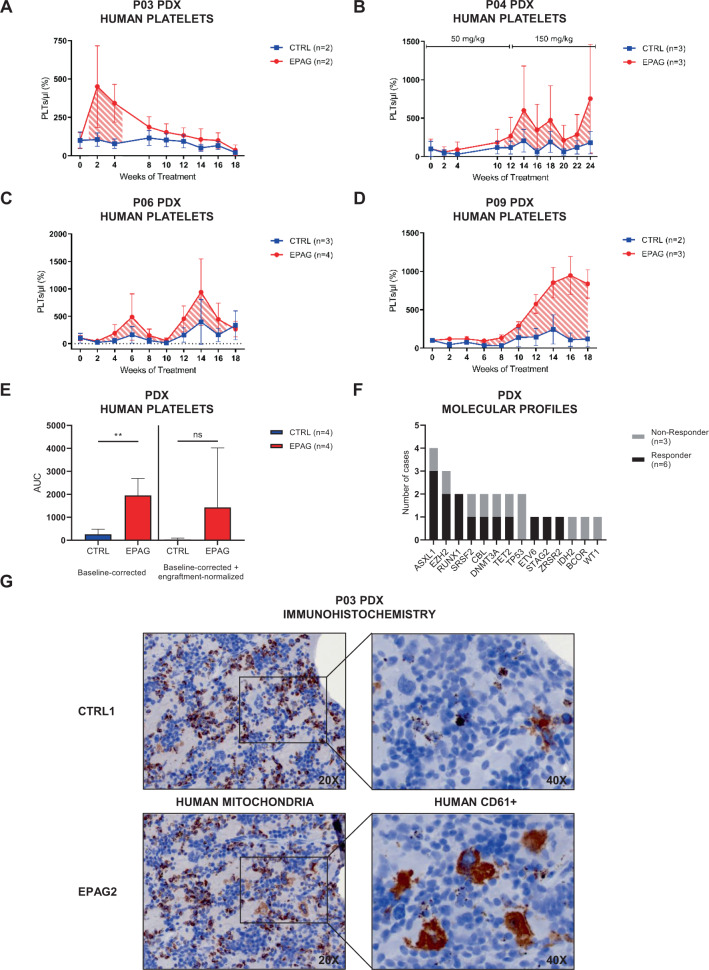
Increased thrombo- and megakaryopoiesis in EPAG-treated MDS patient-derived xenografts. **A**–**D** Mean baseline-corrected number of human platelets (PLTs) in the course of 18–24 weeks of treatment for groups of eltrombopag- (EPAG, red) and vehicle-treated (CTRL, blue) xenografted mice of P03, P04, P06, and P09. Striped areas indicate response to EPAG defined as two-fold change of PLT production. Initial number of PLTs for each mouse was taken as individual baseline. Peripheral blood (PB) was sampled biweekly, unless otherwise indicated. The absolute number of human PLTs per microliter PB was calculated in relation to the number of beads recorded. For P04, dose was escalated after 12 weeks from 50 to 150 mg/kg for additional 12 weeks. See also Supplementary Fig. S4A, B for data on additional cases. **E** Area under the curve (AUC) values for graphs **A**–**D** and mean baseline-corrected engraftment-normalized data (not shown). Data were analyzed using unpaired, two-tailed *t*-test. **F** Comparison of MDS-related mutations between xenograft non-responder and responder. See also Supplementary Fig. S4H for data on cytogenetic aberrations. **G** Serial paraffin sections from CTRL1 and EPAG2 of P03 were stained for human mitochondria and CD45+ CD61+ megakaryocytes using immunohistochemistry (magnification ×20 and ×40). Data in **A**–**E** are represented as mean ± SD. **p < 0.01.

In addition to patient P01, mean baseline-corrected results from PDX of P03, P04, P06, and P09 showed clear differences in PLT counts in PB between groups of treatment (Fig. 3A–D). Response was defined as two-fold increase of PLT production as compared to untreated xenografts. In summary, the total area under the curve of baseline-corrected absolute PLT levels was significantly higher for the EPAG-treated groups as compared to untreated CTRLs (Fig. 3E). This result was still retained after further normalization to the corresponding engraftment rates at the 6-weekly BMA time points, albeit not with statistical significance possibly due to fewer data points (Fig. 3E). Interestingly, in case of P04, administration of the standard EPAG dose from week 0–12 resulted only in a marginal increase in PLTs, whereas subsequent dose escalation of 50–150 mg/kg finally triggered a strong response in all *n* = 3 EPAG-treated xenografts (Fig. 3B). The three patients P02, P05, and P07 were non-responders, because either no human PLTs could be detected at any time point (P02 and P07) or PLT values showed similar profiles for EPAG vs. CTRL (P05) (Supplementary Fig. S4A). Overall, response to EPAG among patients differed regarding its onset and duration, but also between single xenografts of the same patient as shown for P08 (Supplementary Fig. S4B). Murine CD41+ PLT levels of all PDX were unaffected by EPAG treatment (Supplementary Fig. S4C) as previously reported [22]. Conversely, we also measured endogenous murine Tpo levels by enzyme-linked immunosorbent assay showing that these were not connected to human PLT production (Supplementary Fig. S4D–F).

To assess whether response to EPAG could be predicted by clinical markers, we evaluated endogenous human Tpo levels in patient BM plasma, molecular profiles of PDX, and International Prognostic Scoring System–revised (IPSS-R) of responders vs. non-responders (Fig. 3F and Supplementary Fig. S4G–J). However, none of these parameters had significant predictive value, possibly due to the small cohort size. Notably, both cases with *TP53* mutations were non-responders with very high IPSS-R classification.

As MDS is frequently associated with deregulated inflammatory signaling profiles in the BM [23], we also performed reverse transcriptase-quantitative PCR analyses to interrogate the expression levels of the inflammation-associated genes *S100A8*, *S100A9*, *CASP1*, and *NLRP3* in primary patient and PDX samples (Supplementary Fig. S5), to evaluate their predictive value for EPAG response. In the primary patient MNCs, expression values were highly heterogeneous and not significantly different between responders vs. non-responders (Supplementary Fig. S5A). Interestingly, when comparing the PDX endpoint data to the primary patient samples and PDX values at 12 weeks post intrafemoral transplantation, expression levels of all genes tended to be elevated (Supplementary Fig. S5B–F). However, this observation was clearly independent of EPAG treatment.

To confirm human engraftment and stimulation of human megakaryopoiesis by EPAG in the PDX model, we performed immunohistochemistry of human mitochondria and hCD61+ in serial sections of PDX tibia from CTRL1 and EPAG2 of patient P03 (Fig. 3G). This confirmed engraftment of human cells and showed a clear increase of human megakaryocytes in the EPAG-treated PDX.

**Fig. 4:**
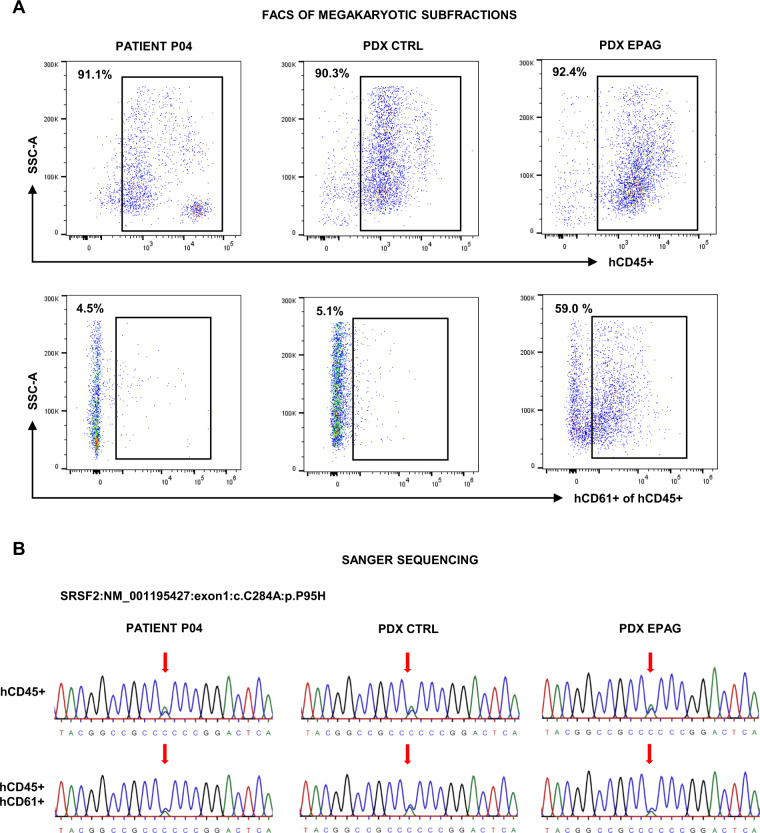
EPAG stimulates human CD45+ CD61+ MDS and not residual healthy megakaryopoiesis in patient-derived xenografts. **A** FACS plots showing sorting gates and percentage (top left corner) of human CD45+ (hCD45+) and hCD45+ hCD61+ cells in the mononuclear cells of patient P04 and pooled bone marrow samples from *n* = 3 CTRL and *n* = 3 EPAG patient-derived xenografts (PDX) at endpoint. **B** Sanger sequencing results of PCR-amplified DNA from sorted hCD45+ and hCD45+ hCD61+ cells of patient P04, as well as CTRL and EPAG PDX for the patient’s MDS-specific mutation *SRSF2*^P95H^. Red arrow indicates the side of mutation where cytosine (C, blue) was replaced by adenine (A, green). Results for the remaining patient–individual mutations are shown in Supplementary Fig. S6A–C. SSC-A, side scatter area.

Finally, we addressed the question whether the observed human PLT production stimulated by EPAG derived from mutated MDS clones or was possibly produced by residual healthy “non-clonal” megakaryopoiesis. We therefore sorted hCD45+ hCD61+ cells from patient P04’s primary MNCs and pooled BM samples of its *n* = 3 CTRL and *n* = 3 EPAG PDX (Fig. 4A). The percentage of hCD45+ hCD61+ cells in the EPAG PDX was ~12-fold higher as compared to the patient’s MNCs and the CTRL group (59.0% vs. 4.5% and 5.1%, respectively), further confirming EPAG’s efficacy. DNA from sorted hCD45+ hCD61+ and hCD45+ cells was used to perform Sanger sequencing of the patient’s four MDS-specific mutations *ASXL1*^Q561X^, *RUNX1*^T196fs^, *SRSF2*^P95H^, and *TET2*^H1904Q^. These analyses revealed that all mutations were readily detectable in the sorted hCD45+ hCD61+ subfraction (Fig. 4B and Supplementary Fig. S6A–C). This confirmed that EPAG at least partly stimulated megakarypoiesis from mutated MDS clones.

### EPAG treatment does not induce disease progression or transformation in vehicle-controlled PDX experiments

**Fig. 5:**
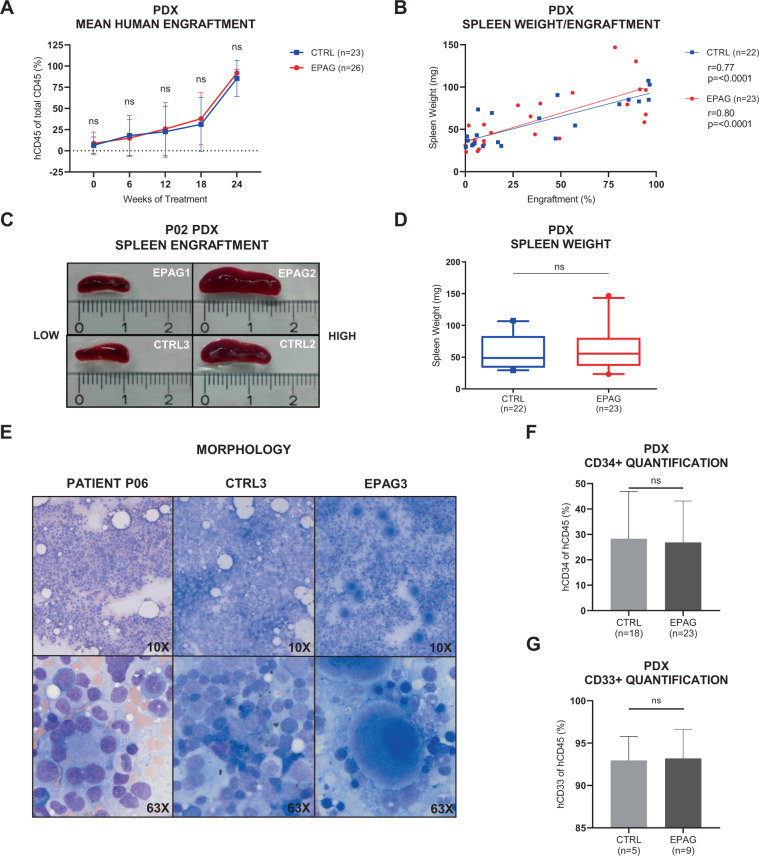
EPAG treatment does not promote progression or transformation in MDS patient-derived xenografts compared to vehicle control. **A** Human engraftment in the bone marrow (BM) for groups of *n* = 26 eltrombopag- (EPAG, red) and *n* = 23 vehicle-treated (CTRL, blue) patient-derived xenografts (PDX) in the course of 18–24 weeks of treatment. Engraftment was assessed every 6 weeks. Individual results for each patient are shown in Supplementary Fig. S7A–H. **B** Correlation between engraftment and spleen weight in PDX of CTRL (*r* = 0.77, *p* = <0.0001) and EPAG group (*r* = 0.80, *p* = <0.0001), respectively. Linear regression analysis was performed using Prism 8 (GraphPad Software) and Spearman’s correlation coefficient was computed. See also Supplementary Fig. S7I for data on correlation between engraftment and spleen size. **C** Pictures of spleens from patient P02’s PDX with low (EPAG1+CTRL3, left) and high engraftment (EPAG2+CTRL2, right). **D** Spleen weight of all PDX from all patients for groups of *n* = 23 EPAG and *n* = 22 vehicle treatment. Data were analyzed using two-tailed Mann–Whitney *U*-test. Whiskers indicate 5–95% percentile. See also Supplementary Fig. S7J for data on spleen size. **E** BM smears of patient P06 (left) and its PDX CTRL3 (middle) and EPAG3 (right) at endpoint stained with May–Grünwald–Giemsa stain (magnification ×10 and ×63). **F** Percentage of human CD45+ CD34 cells in the BM of *n* = 23 EPAG- and *n* = 18 vehicle-treated PDX at endpoint. **G** Percentage of human CD45+CD33 cells in the BM of *n* = 9 EPAG- and *n* = 5 vehicle-treated PDX at endpoint. Data in **A**, **F**, and **G** are represented as mean ± SD. ns, not significant.

The main aim of this study was to use the advantage of a vehicle-controlled experimental PDX model to investigate whether EPAG may have intrinsic disease-propagating effects. To this end, we first compared engraftment rates in dependency of treatment. There was no significant difference regarding mean percentage of hCD45+ cells between CTRL (*n* = 23) and EPAG (*n* = 26) PDX groups (Fig. 5A). Individual results for each patient are shown in Supplementary Fig. S7A–H. As a potential marker for extramedullary hematopoiesis, we also compared spleen weights and sizes of all PDX. Although spleen weight and size clearly correlated with total engraftment (Fig. 5B, C and Supplementary Fig. S7I), there was no difference between EPAG or vehicle treatment (Fig. 5D and Supplementary Fig. S7J). Finally, BM smears of xenografts were morphologically evaluated for the presence of blasts by a board certified hematopathologist. Although EPAG-treated samples recurrently presented with clearly increased numbers of megakaryocytes, blasts were not detectable in either of the treatment groups. As an example, Fig. 5E shows the BM smears of patient P06, and its xenografts CTRL3 and EPAG3. These results were also corroborated by flow cytometry analyses, which revealed no increase in the relative burden of hCD45+ hCD34+ or hCD45+ hCD33+ cells in the EPAG-treated mice (Fig. 5F, G). Importantly, this was also true for mouse EPAG3 of patient P04 (Supplementary Fig. S7K), which had to be killed after 20 weeks of treatment due to weight loss.

### EPAG treatment does not promote clonal evolution in patient–individual xenografts compared to vehicle CTRL

The PDX experimental setup of this study for the first time enabled long-term evaluation of MDS-specific clonal evolution in dependency of EPAG treatment under vehicle-controlled conditions on a patient–individual level.

**Fig. 6:**
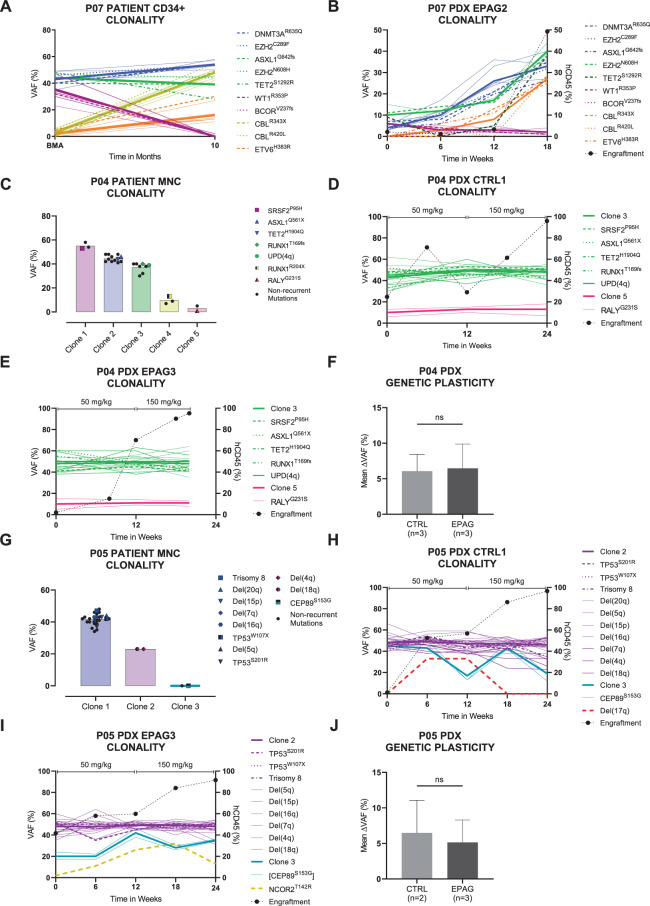
EPAG treatment does not affect clonal evolution in MDS patient-derived xenografts compared to vehicle control. Bone marrow clonality of patients and exemplary xenografts during 18–24 weeks of experiment reconstructed by clustering of variant allele frequencies (VAFs) using the bioinformatic tool SciClone. Mutations within the same clone are equally colored. Thicker lines represent the mean clonal VAF. Non-synonymous mutations are displayed with superscripted amino acid change. Dotted black line describes the course of human engraftment. See also Supplementary Fig. S8A–Z for additional data. **A**, **B** VAFs of patient P07’s CD34+ cells in the course of 10 months and human CD45+ (hCD45+) cells from EPAG2. **C**–**E** VAFs of patient P04’s mononuclear cells (MNCs) and hCD45+ cells from CTRL1 and EPAG3. Dose was escalated after 12 weeks from 50 to 150 mg/kg. EPAG3 had to be eliminated after 20 weeks due to excessive weight loss. **F** Total mean deltaVAF of P04’s *n* = 3 CTRL and *n* = 3 EPAG xenografts. **G**–**I** VAFs of patient P05’s MNCs and hCD45+ cells from CTRL1 and EPAG3. Dose was escalated after 12 weeks from 50 to 150 mg/kg. **J** Total mean deltaVAF of P05’s *n* = 2 CTRL and *n* = 3 EPAG xenografts. Data in **F** and **J** were analyzed using unpaired, two-tailed *t*-test and is represented as mean ± SD. ns, not significant; EPAG, eltrombopag; BMA, bone marrow aspiration.

Consequently, we were able to obtain informative series of clonal composition in xenografts inferred from WES of as many as five serial time points during an experimental duration of up to 24 weeks for P01 (Fig. 2E–G), P03, P04, P05, P07, P08, and P09 (Fig. 6A–J and Supplementary Fig. S8A–X). Stand-alone endpoint data were acquired for P02 and P06 (Supplementary Fig. S8Y, Z). Different molecularly defined clones present in the primary patient and PDX samples were grouped into coherent mutational and cytogenetic clusters using the R tool SciClone (Fig. 1E). This resulted in the detection of three to five separable dominant and subclones per patient. In the majority of cases, the MDS PDX carried a similar mutational profile as their input patient sample (Supplementary Fig. S9).

Data from P07 served as important proof-of-principle case. For this patient, we had primary CD34+ cells of two consecutive clinical time points available, as well as serial samples of one single PDX (EPAG2), which was treated with EPAG. In the initial patient sample, five subclones defined by ten different MDS-typical mutations could be identified (Fig. 6A). Over a time span of 10 months, natural clonal evolution occurred in this patient, consisting of the replacement of an intermediate clone carrying a *WT1*^R353P^ and *BCOR*^V237fs^ mutation by a new clone with two *CBL* mutations and an additional *ETV6*^H383R^ mutation. Interestingly, this change of clonal composition was almost identically reproduced in the corresponding EPAG-treated xenograft within a time period of 18 weeks (Fig. 6B), which confirmed that our PDX model can faithfully recapitulate a natural course of clonal evolution and further that this progress was not caused by EPAG treatment.

The cases P01, P03, P04, P05, P08, and P09 enabled standardized comparisons of clonal composition between groups treated with either EPAG or vehicle CTRL. Although the data were highly individual on a per case basis, in summary, we could not detect indicators for a systematic or recurrent difference of clonal selection or burden induced by EPAG (Figs. 2E–G and 6A–J, and Supplementary Fig. S8A–X). The same was true for P02 and P06, of which single endpoint data sets were available (Supplementary Fig. S8Y, Z).

For patients P04 and P05, data of all PDX showed that their clonal composition remained stable throughout the whole experiment even after EPAG dose escalation from 50 to 150 mg/kg after 12 weeks of treatment for a total treatment time of 24 weeks (Fig. 6C–J and Supplementary Fig. S8G–M). Importantly, this was also true for mouse EPAG3 of patient P04, which had to be killed earlier.

Of note, single xenografts of P01, P02, P05, and P08 displayed (transient) emergence and decline of novel molecular lesions (Figs. 2G and 6H, I, and Supplementary Figs. S8B and S7O, P). Nevertheless, this did not influence the overall notion of stable clonal composition in both conditions, which was further corroborated by comparable overall stable dynamics of the deltaVAFs (Figs. 2I and 6F, J, and Supplementary Fig. S8F, Q, X).

## Discussion

Tpo receptor agonists such as EPAG have been approved as efficacious and well-tolerated drugs for non-malignant disorders associated with thrombocytopenia, as well as severe aplastic anemia. Although several clinical trials were already able to demonstrate efficacy and tolerability of EPAG in MDS patients, concerns about a possible promotion of disease progression could not be ruled out entirely. This is owed to both the intrinsic characteristics of MDS being a clinically and molecularly highly heterogeneous disease and the limits of clinical trials in humans, which cannot test study drug and placebo on the same patient.

To this end, PDX models represent a highly promising approach to foster improved performance of precision medicine for cancer treatment [24]. By reproducing a patient’s disease in multiple animals and allowing for patient–individual substance testing, these platforms offer unique advantages [5]. Although MDS PDX models have been established as proof of principle, they have not been applied as systematic platforms for preclinical substance testing in MDS yet. Although MDS PDX-based testing of chemotherapeutic agents, antibodies, and different inhibitors has been demonstrated in some reports, these were of anecdotal scope, limited by low patient and xenograft numbers, short treatment durations, or the absence of vehicle CTRLs [25–28]. In this study, we performed a systematical preclinical evaluation of EPAG regarding its efficacy and influence on the clonal BM composition in *n* = 49 xenografts derived from *n* = 9 MDS patients with thrombocytopenia. By generating multiple xenografts of each patient and dividing them into treatment groups of EPAG and vehicle, substance-specific and disease-related effects could be distinguished. Using flow cytometric analysis and WES, clinical parameters such as human PLT counts and the molecular clonal BM composition could be monitored with high reliability in vivo. A treatment duration of up to 24 weeks allowed for multiple molecular follow-ups, which were mandatory for tracing clonal evolution. Importantly, as high MDS engraftment rates of over 90% did not affect the general health of the mice, even longer experiments seem to be possible.

EPAG was proven to be consistently efficacious in a group of *n* = 5 MDS patient xenografts, whereas *n* = 3 cases were non-responders. Case P08 presented with a heterogeneous response to EPAG as only one out of *n* = 3 PDX showed elevated levels of human PLTs. This corresponds to an overall response rate of 56%, which was higher as compared to real-life clinical trials where the EPAG’s efficacy was demonstrated in 24–47% of cases [12–15]. The specificity of response in our xenografts was corroborated by the fact that patient P04 responded to EPAG only after dose escalation from 50 to 150 mg/kg, despite 12 weeks of previous treatment with the lower dosage. Of note, patient P07 had been treated with EPAG clinically and failed to respond similarly to its corresponding PDX, which was also a non-responder, and therefore faithfully recapitulated the real-life clinical outcome. Our cohort was not powered to yield predictive molecular markers of response. However, interestingly, both MDS patients who carried *TP53* mutations were non-responders and were classified with very high risk according to the IPSS-R. This could be reflective of the notion that EPAG treatment may not be suitable for high-risk MDS patients with advanced disease. *TP53* mutations are increasingly emerging as the utmost candidate lesions for a particularly adverse prognosis in MDS [29, 30]. Moreover, although little data are available, there is evidence that *TP53* mutations interfere with megakaryocytic differentiation, e.g., also in the context of lenalidomide resistance [31]. In light of this, it seems remarkable that even these two very high-risk cases were not subject to transformation under EPAG stimulation.

The most important question to answer was whether EPAG promotes disease transformation of MDS. Examination of PDX BM smears did not show increased numbers of blasts or any signs of morphological transformation in both the EPAG- and vehicle-treated groups. In addition, flow cytometry analysis showed no significant differences between the two conditions regarding human engraftment and percentage of hCD45+ CD34+ cells in the BM. Finally, highly comprehensive molecular analysis by WES of hCD45+ BM cells from serial time points was performed. Importantly, there was no evidence for a higher risk of clonal evolution in EPAG-treated MDS xenografts as compared to CTRLs. In contrast, when comparing the genetic plasticity by assessing the total mean deltaVAF, it tended to be lower in EPAG PDX. However, in single cases, we could detect dynamic, partly transient clonal shifts, e.g., by the emergence of novel molecular lesions in *n* = 4 (17%) vehicle- and *n* = 2 (8%) EPAG-treated xenografts. This proves that an experimental time frame of 18–24 weeks is sufficient for clonal expansion and/or displacement, and the acquisition of additional MDS-related mutations, and therefore to observe potential clonal changes by treatment.

In conclusion, the main point learned from our experimental setup is that we were able to perform controlled EPAG vs. vehicle experiments within primary samples from the same MDS patient. This is something, which is not possible in a real-life clinical trial. On the background of existing clinical trial data on EPAG, which has been repeatedly borderline or weak in ruling out drug-intrinsic disease-transforming effects, the opportunity to test this in our MDS PDX therefore added an additional layer of safety information to the pre-existing data. This additional confirmation of safety should encourage future studies to test EPAG in combination with other substances supporting hematopoiesis or aid hematopoietic recovery after hematotoxic treatment. For example, combination of EPAG with luspatercept could possibly synergistically augment their supportive effects in pancytopenic MDS patients. Finally, another point learned from our data is that it is strongly advisable to accompany or precede clinical trials in humans with preclinical substance testing using PDX models as presented in this study.

## Supplementary information

Supplementary Data
