# Supplementary material for: Preclinical evaluation of eltrombopag in a PDX model of myelodysplastic syndromes
Source: Leukemia. 2021 Jun 25;36(1):236–47. doi: 10.1038/s41375-021-01327-w (PMC8727300; doi:10.1038/s41375-021-01327-w)
Supplement: Supplementary file 1 — Supplementary Data [file 41375_2021_1327_MOESM1_ESM.pdf]

Supplementary Methods

WES and bioinformatic analysis

DNA of primary patient and patient-derived xenograft (PDX) samples was isolated using the AllPrep DNA/RNA Kit (Qiagen, Hilden, Germany). For WES, 250 ng of high molecular genomic DNA from patient and PDX samples was subjected to the Nextera DNA Flex Kit (Illumina, San Diego, CA, USA) and subsequent hybrid capture using the xGen Exome Research Panel (IDT, Coralville, IA, USA). For PDX samples with less DNA available, PCR cycles during capture were increased according to the manufacturer’s protocol. The final library pools were sequenced on a S4 Nova Seq Flow Cell (Illumina) with 150 bp PE. For mutational calling, raw sequencing data was subjected to a previously established bioinformatical pipeline [1] including fastq-trimming (trimmomatic v0.39), alignment (bwa v0.7.9) and PCR deduplication (picard MarkDuplicates v2.20.5). BAM files were realigned and recalibrated with the gatk bundle (v3.8). Potentially overlapping forward and reverse reads were softclipped using bamUtil clipOverlap (v1.0.14). After independent mapping to hg19 and mm10Bam, files from PDX samples were filtered for contaminating mouse reads using the R package xenofilteR [2]. Somatic mutations were called by gatk Mutect2 (gatk v4.1.3.0). DNA from matched MSCs served as germline controls. Only variants passing FilterMutectCalls were further annotated with annovar [3] and subsequently verified by manually visualization in IG Viewer. For homogenous comparison among multiple PDX from a single patient, each tumor:normal comparison was called independently in this manner. Subsequently, all passed filter sites from all PDX were combined (gatk genotypeMergeOptions UNIQUIFY) and re-quantified including the primary bone marrow (BM) sample using VarScan (v2.4.4) mpileup2snp and mpileup2indel, respectively. For detection of copy number and LOH events in primary and PDX samples, the R package Sequenza [4] was used. For subsequent quantification of these aberrations, averaged B allele frequency of heterozygous SNPs was used. On average, a mean coverage of 89.8 fold was achieved for patient and PDX samples. For tracking of subclonal populations and their evolution, individual clustering of VAFs for consecutive samples of each PDX and the corresponding initially transplanted patient sample was performed using the R package SciClone (v1.1) [5]. Data can be accessed via European Genome-phenome Archive (EGA, https://ega-archive.org/) using the identifier EGAS00001005329.

Code availability

All tools used in this study are available on GitHub:

- Trimmomatic: https://github.com/usadellab/Trimmomatic
- Burrows-Wheeler Aligner (BWA): https://github.com/lh3/bwa
- Picard: https://github.com/broadinstitute/picard
- Genome Analysis Toolkit (GATK): https://github.com/broadinstitute/gatk
- BamUtil: https://github.com/statgen/bamUtil
- XenofilteR: https://github.com/PeeperLab/XenofilteR
- IG Viewer: https://github.com/igvteam/igv
- VarScan: https://github.com/dkoboldt/varscan
- Sequenza: https://github.com/cran/sequenza
- SciClone https://github.com/genome/sciclone

May-Grünwald-Giemsa stain

BM smears of PDX prepared from tibiae at endpoint were fixed with methanol for 10 min. For diluting and washing, pH 6.8-buffered destilled water (dH2O) (Merck, Darmstadt, Germany) was used. Staining was performed as follows: 7 min 1+1 May-Grünwald solution (Merck), 20 min 1+6 Giemsa solution (Merck), 10 sec dH2O, 4 min dH2O and 4 min dH2O. Images were acquired using an PreciPoint M8 microscope and scanner (PreciPoint, Friesing, Germany).

Immunohistochemistry

Tibias of PDX were fixed in 10% formaldehyde for 48 h and subsequently decalcified in EDTA. Following, bones were embedded in paraffin and cut into 10 µm sections. After deparaffinization, sections were stained using the anti-human mitochondria antibody (MAB1273; Merck) and anti-human CD61 (CI869C002; DCS Diagnostics, Hamburg, Germany), and detected with the EnVision Detection Systems Peroxidase/DAB, Rabbit/Mouse (Agilent, Santa Clara, CA, USA) and the OptiView DAB Detection Kit (Roche, Basel, Switzerland), respectively. To exclude crossreactivity of the antibodies with mouse tissue, tibia sections of a mouse without human transplant were stained beforehand. Finally, the PDX slides were counter-stained with hematoxylin. Images were acquired using an PreciPoint M8 microscope and scanner (PreciPoint).

Mouse Tpo ELISA

Peripheral blood (PB) from PDX was sampled bi-weekly starting from 12 weeks post intrafemoral transplantation. Lysed PB was centrifuged for 15 min at 2000 x g and 4 °C to generate plasma. Plasma was stored at -80 °C until further use. Mouse Tpo levels in PDX plasma were determined at week 0, 4/6, 12 and 18 of experiment using the Mouse Thrombopoietin Quantikine ELISA Kit (R&D Systems, Minneapolis, MN, USA) according to the manufacturer's protocol. Colorimetric signals were measured using a Tecan Infinite 200 PRO 8 (Tecan Group Ltd., Männedorf, Switzerland) plate reader.

Human Tpo ELISA

MDS patient BM plasma was prepared from samples of diagnostic BM aspirations after informed written consent and stored at -80 °C until further use. Human Tpo levels in patient BM samples were determined using the Human Thrombopoietin Quantikine ELISA Kit (R&D Systems). Colorimetric signals were measured using a Tecan Infinite 200 PRO 8 (Tecan Group Ltd.) plate reader.

RT-qPCR analysis

RNA of primary patient and PDX samples was isolated using the AllPrep DNA/RNA Kit (Qiagen). For cDNA synthesis, the QuantiTect Reverse Transcription Kit (Qiagen) was used according to the manufacturer's protocol. Used primer sequences are listed in **Supplementary Table S2**. For RT-qPCR, samples were assayed in triplicates using the LightCycler® 480 SYBR Green I Master reaction mix (Roche) and a LightCycler® 480 II (Roche). For analysis, the delta-delta Ct method was performed.

Supplementary References

1. Mossner M, Jann J-C, Wittig J, Nolte F, Fey S, Nowak V *et al.* Mutational hierarchies in myelodysplastic syndromes dynamically adapt and evolve upon therapy response and failure. *Blood* 2016; **128**: 1246–1259.
2. Jo S-Y, Kim E, Kim S. Impact of mouse contamination in genomic profiling of patient-derived models and best practice for robust analysis. *Genome Biol* 2019; **20**: 231.
3. Wang K, Li M, Hakonarson H. ANNOVAR: functional annotation of genetic variants from high-throughput sequencing data. *Nucleic Acids Res* 2010; **38**: e164–e164.
4. Favero F, Joshi T, Marquard AM, Birkbak NJ, Krzystanek M, Li Q *et al.* Sequenza: allele-specific copy number and mutation profiles from tumor sequencing data. *Ann Oncol* 2015; **26**: 64–70.
5. Miller CA, White BS, Dees ND, Griffith M, Welch JS, Griffith OL *et al.* SciClone: Inferring Clonal Architecture and Tracking the Spatial and Temporal Patterns of Tumor Evolution. *Plos Comput Biol* 2014; **10**: e1003665.

Supplementary Table S1

Supplementary Table S1 Flow cytometric antibodies used in this study.

Antigen	Fluorochrome	Clone	Distributor	Cat. No.
human CD3	PerCP-Cy5.5	UCHT1	BD Biosciences	560835
human CD19	FITC	HIB19	BD Biosciences	555412
human CD33	APC	WM53	Biolegend	303407
human CD34	FITC	561	Biolegend	343603
human CD41	PE	HIP8	Biolegend	303705
mouse CD41	APC/Cy7	MWReg30	Biolegend	133927
human CD45	PE	HI30	BD Biosciences	560975
mouse CD45	APC/Cy7	30-F11	Biolegend	103115
human CD61	APC	VI-PL2	Biolegend	336411

BD Biosciences, San Jose, CA, USA; Biolegend, San Diego, CA, USA

Supplementary Table S2

Supplementary Table S2 Primer sequences for RT-qPCR and standard PCR used in this study.

Gene	Strand	Sequence (5‘→3‘)	Product size
S100A8	Forward	TGTCTTTCAGAAGACCTGGTGG	199 bp
	Reverse	GACGTCTGCACCCTTTTTCC	
S100A9	Forward	CTGCAAAATTTTCTCAAGAAGGAGA	160 bp
	Reverse	CACCCTCGTGCATCTTCTCG	
CASP1	Forward	GAAAAGCCATGGCCGACAAG	189 bp
	Reverse	CGGAGTCAATCAAAGCTCGG	
NLPR3	Forward	ATGTGGGGGAGAATGCCTTG	199 bp
	Reverse	TTGTCTCCGAGAGTGTTGCC	
GUSB	Forward	CCTGCGTCCCACCTAGAATC	188 bp
	Reverse	AGGTGCCCGTAGTCGTGATA	
ASXL1 (c.C1681T)	Forward	GTTGAGTCTGTGGCTTCTCG	432 bp
	Reverse	TCTCACTAAACTTGAAGGCTGTC	
RUNX1 (c.587_588del)	Forward	GCCAAAATTCCGGGAGTGTT	510 bp
	Reverse	TGCTGCCCTGAATGGTTCTA	
SRSF2 (c.C284A)	Forward	GGCCGCCACTCAGAGCTA	461 bp
	Reverse	CTTCGCCGCGGACCTTTG	
TET2 (c.T5712A)	Forward	AGGACAACGATGAGGTCTGG	409 bp
	Reverse	GACATGGTCCTTTCGGCAAG	

Supplementary Fig. S1

A

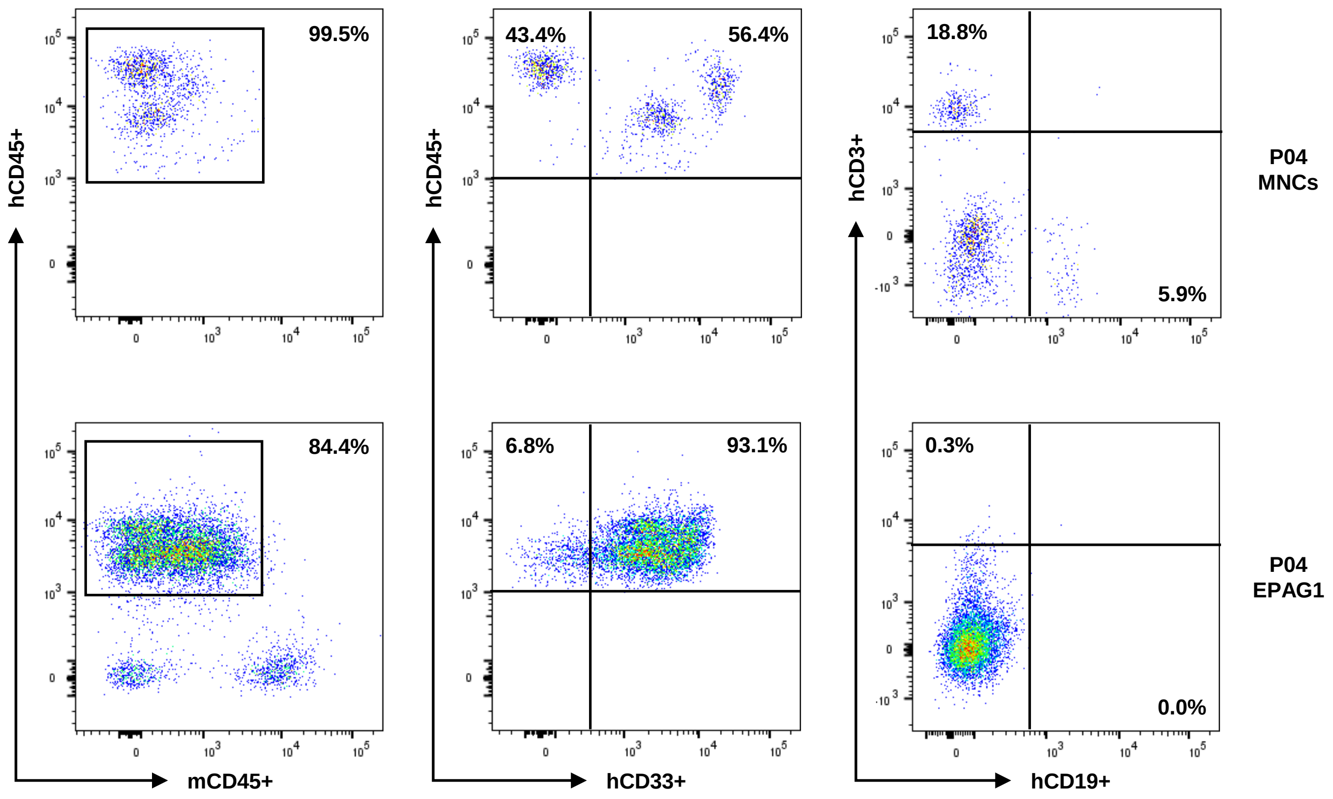

B

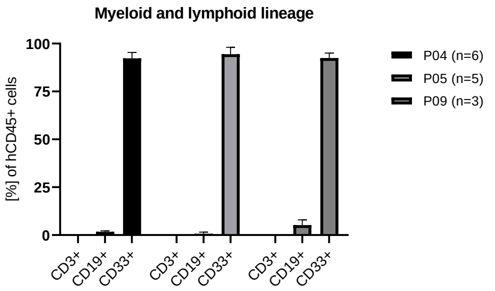

Supplementary Fig. S1 Distribution of myeloid and lymphoid lineage in the xenograft bone marrow. (A) Exemplary flow cytometry plots of patient P04's mononuclear cells (MNCs) and bone marrow (BM) of xenograft EPAG1 at endpoint showing gating strategy to determine percentage of the human myeloid (hCD33+) and lymphoid (hCD3+, hCD19+) cells within the hCD45+ cell population. **(B)** Percentage of hCD33+, hCD3+ and hCD19+ cells in the BM of n=14 xenografts of n=3 patients. Data is represented as means ± SD. mCD45+, murine CD45+

Supplementary Fig. S2

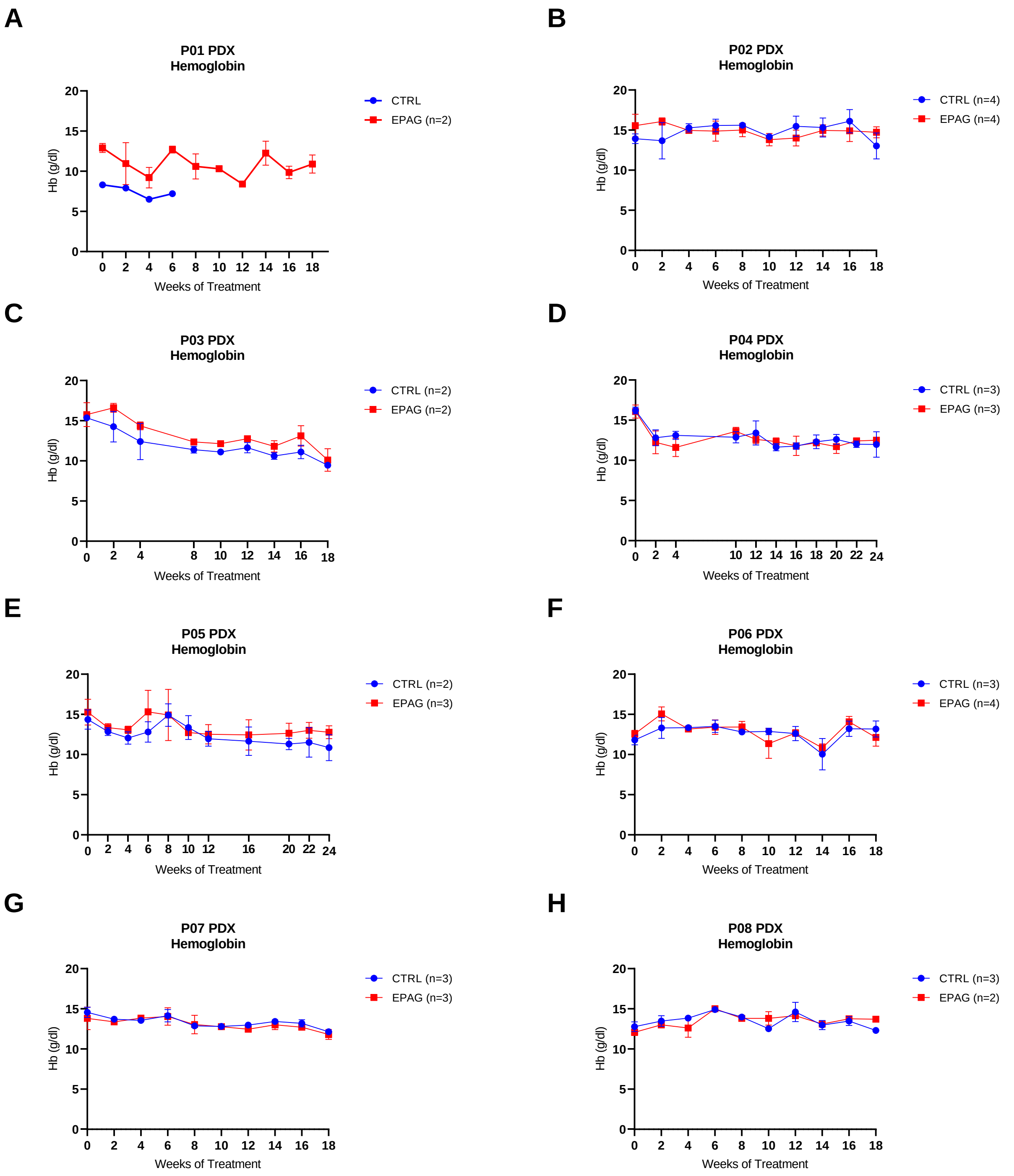

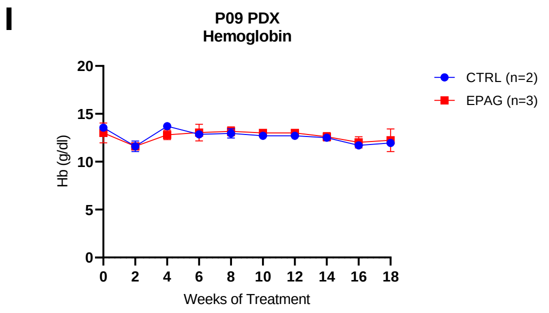

Supplementary Fig. S2 Total hemoglobin (Hb) levels of xenografts in the course of 18-24 weeks of experiment. Peripheral blood from patient-derived xenografts was sampled bi-weekly starting from 12 weeks post intrafemoral transplantation. Total Hb levels (mouse + human Hb) were measured using the hematology analyzer ABX Micros 60 (Horiba, Kyoto, Japan). Data is represented as mean ± SD.

Supplementary Fig. S3

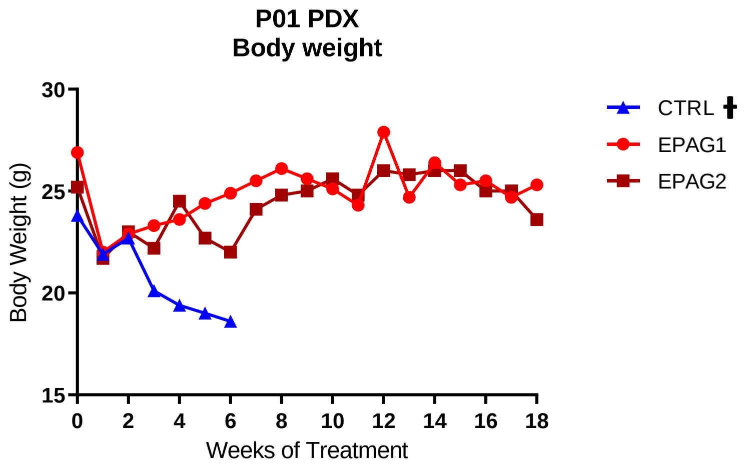

Supplementary Fig. S3 Body weight of patient P01's xenografts throughout experiment. Of n=3 xenografts, n=2 were treated with eltrombopag (EPAG1+2, red) and n=1 received vehicle control (CTRL, blue). CTRL mouse had to be sacrificed six weeks into treatment phase due to excessive weight loss.

Supplementary Fig. S4

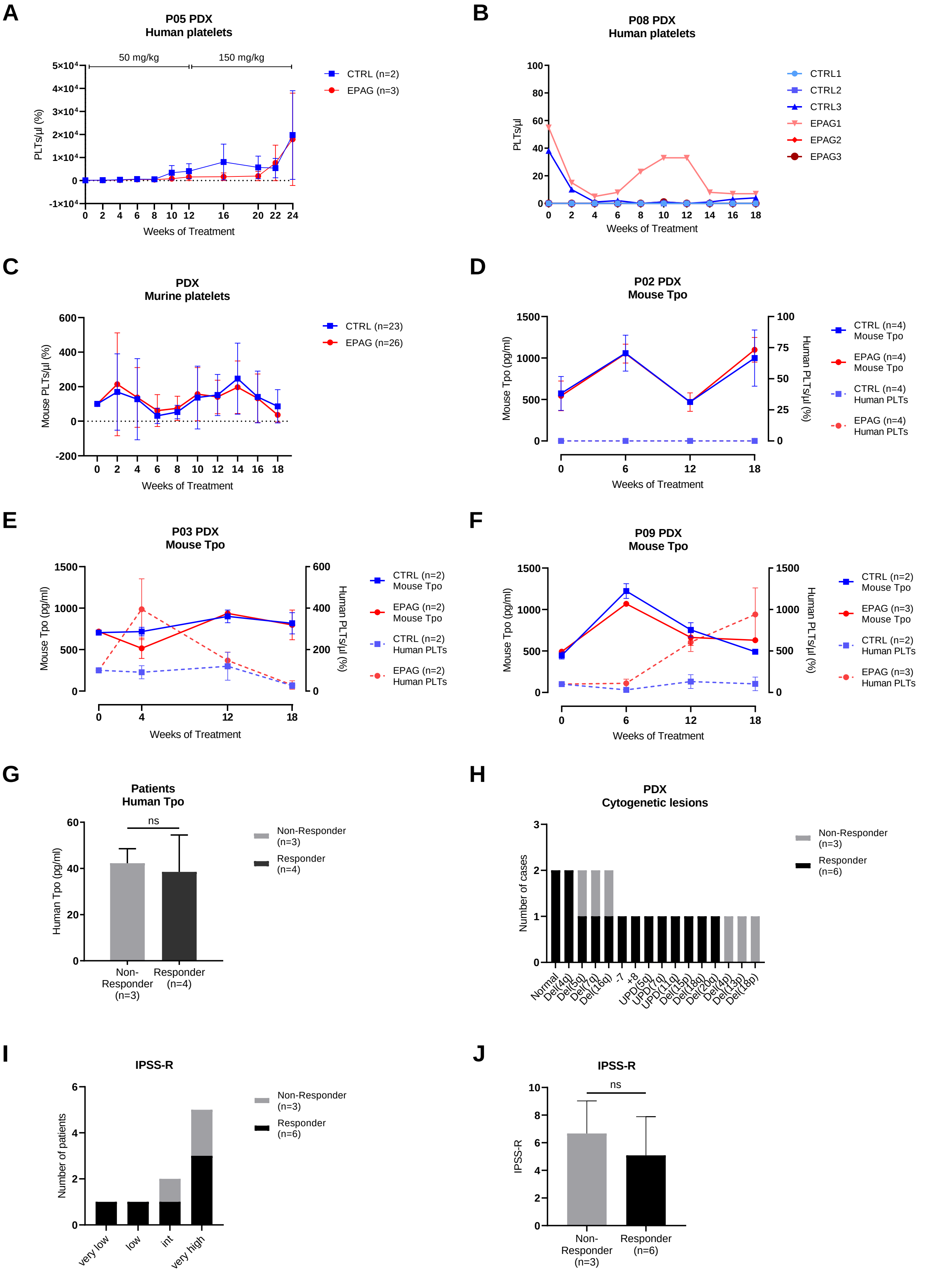

Supplementary Fig. S4 continued

Supplementary Fig. S4 Increased human thrombopoiesis in EPAG-treated xenografts. (A) Mean baseline-corrected number of platelets (PLTs) in the course of 24 weeks of treatment for groups of eltrombopag- (EPAG, red) and vehicle-treated (CTRL, blue) patient-derived xenografts (PDX) of P05. Initial number of PLTs for each PDX was taken as individual baseline. Peripheral blood (PB) was sampled bi-weekly. The absolute number of human PLTs per microliter PB was calculated in relation to the number of beads recorded. Dose was escalated after 12 weeks from 50 mg/kg to 150 mg/kg for additional 12 weeks. **(B)** Individual courses of PLTs of patient P08's for n=3 CTRL and n=3 EPAG PDX throughout 18 weeks of treatment. **(C)** Mean baseline-corrected number of murine PLTs in the course of 18 weeks of treatment for groups of EPAG- (n=26) and vehicle-treated (n=23) PDX. **(D-F)** Murine Tpo levels in PDX plasma (continuous lines) of n=1 non-responder (P02) and n=2 responders (P03, P09) in EPAG and CTRL groups at week 0, 4/6, 12 and 18 of experiment assayed by ELISA. Courses of human PLTs (dotted lines) are shown in comparison. **(G)** Human Tpo levels in n=7 (n=4 responders, n=3 non-responders) patient bone marrow (BM) plasma samples derived from the same time point as the xenografted CD34+ samples. **(H)** Comparison of PDX non-responder and responder regarding distribution of cytogenetic aberrations. **(I)** Distribution of classifications based on IPSS-R from all n=9 MDS patients between non-responder and responder. **(J)** Comparison of IPSS-R between non-responder and responder. Data in **G** and **J** was analyzed using unpaired, two-tailed t-test. Data in **A, C-G** and **J** is represented as mean ± SD. ns, not significant.

Supplementary Fig. S5

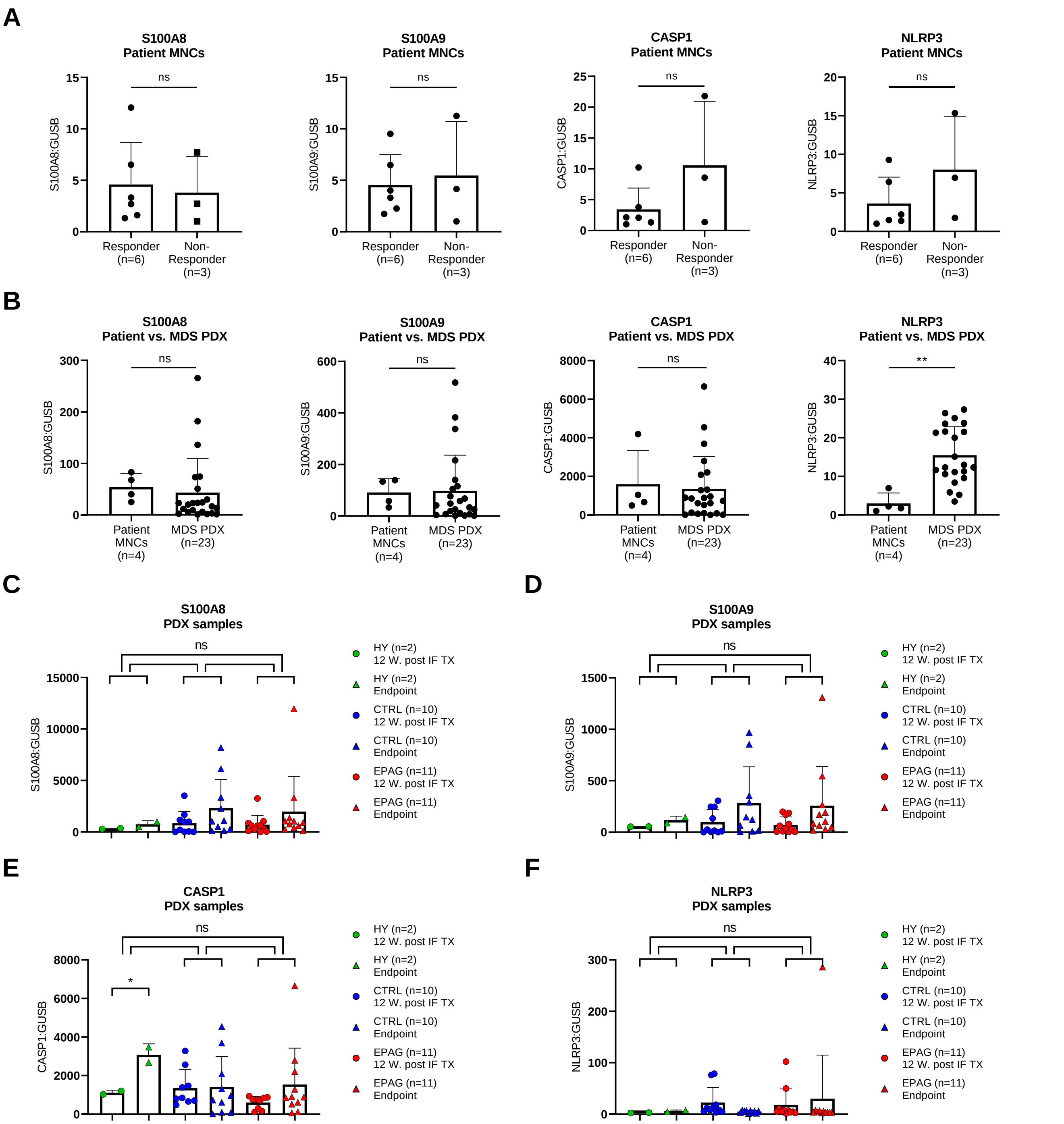

Supplementary Fig. S5 Inflammatory markers are no predictors for response to EPAG nor affected by EPAG treatment. Expression of the inflammation-associated genes S100A8, S100A9, CASP1 and NLRP3 was determined using RT-qPCR. Samples of patient mononuclear cells (MNCs) and patient-derived xenograft (PDX) samples were analyzed separately using the delta-delta Ct method. **(A)** Patient MNC samples of n=6 responder and n=3 non-responder. **(B)** Samples of MNCs from patient P02, P03, P04 and P05 and their respective PDX at endpoint in comparison. **(C-F)** Samples of n=10 vehicle- (CTRL) and n=11 EPAG-treated MDS PDX of P02, P03, P04 and P05, as well as n=2 healthy (HY), untreated PDX at 12 weeks post intrafemoral transplantation (IF TX) and endpoint. Data in **A** and **B** was analyzed using unpaired, two-tailed t-test. Data in **C-F** was analyzed using unpaired one-way ANOVA. All data is represented as mean ± SD. *p<0.05; **p<0.01; ns, not significant; EPAG, eltrombopag..

Supplementary Fig. S6

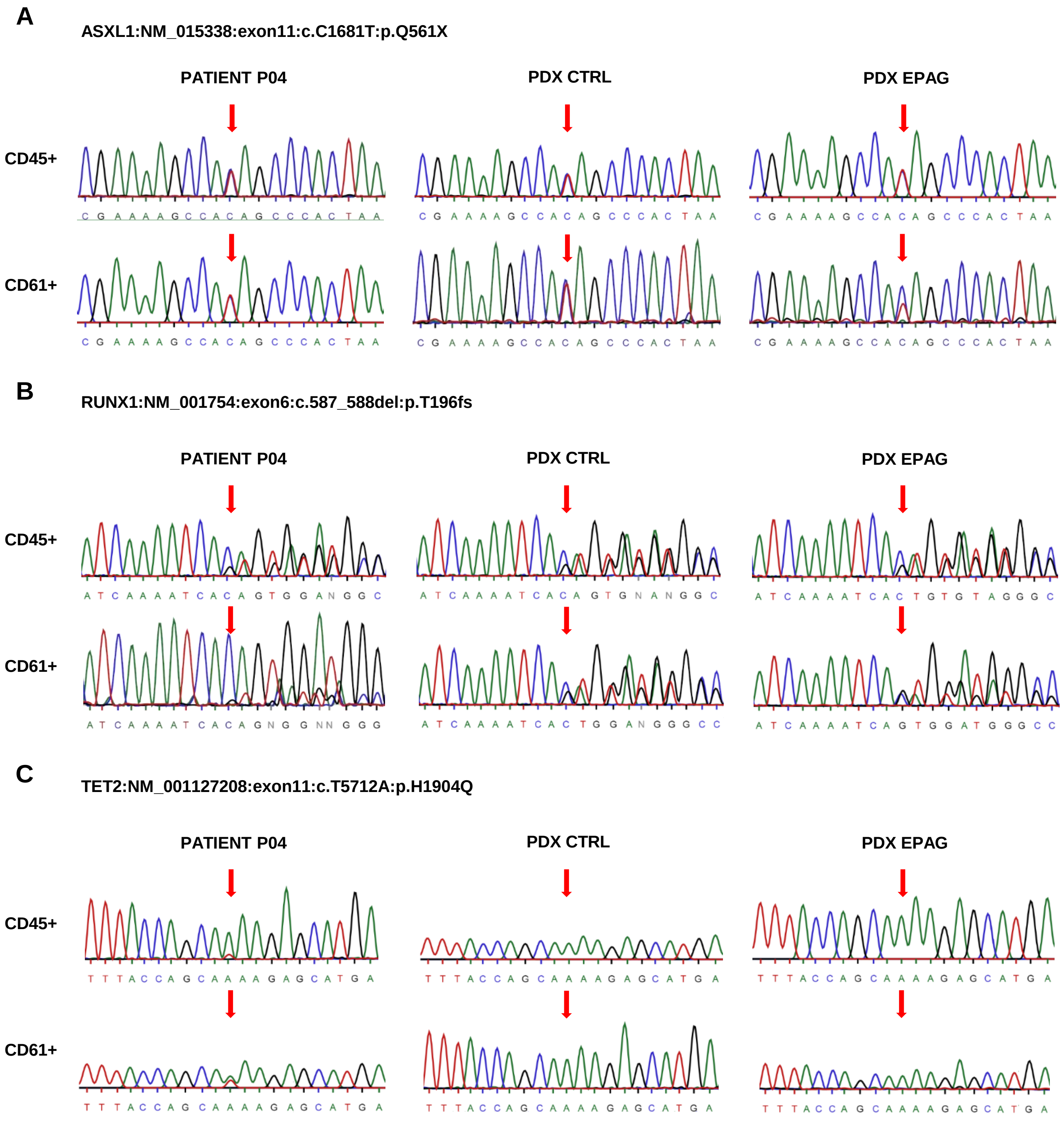

Supplementary Fig. S6 EPAG stimulates human CD45+ CD61+ MDS and not residual healthy megakaryopoiesis in patient-derived xenografts. Sanger sequencing results of PCR-amplified DNA from sorted hCD45+ and hCD45+ hCD61+ cells of patient P04, and CTRL and EPAG PDX for the patient's MDS-specific mutations. **A)** Results for mutation ASXL1^{Q561X}. Red arrow indicates the side of mutation where cytosine (C, blue) was replaced by thymine (T, red). **B)** Results for mutation RUNX1^{T196fs}. Red arrow indicates the side of mutation where T and guanine (G, black) were deleted causing a frame shift. **C)** Results for mutation TET2^{H1904Q}. Red arrow indicates the side of mutation where T was replaced by A.

Supplementary Fig. S7

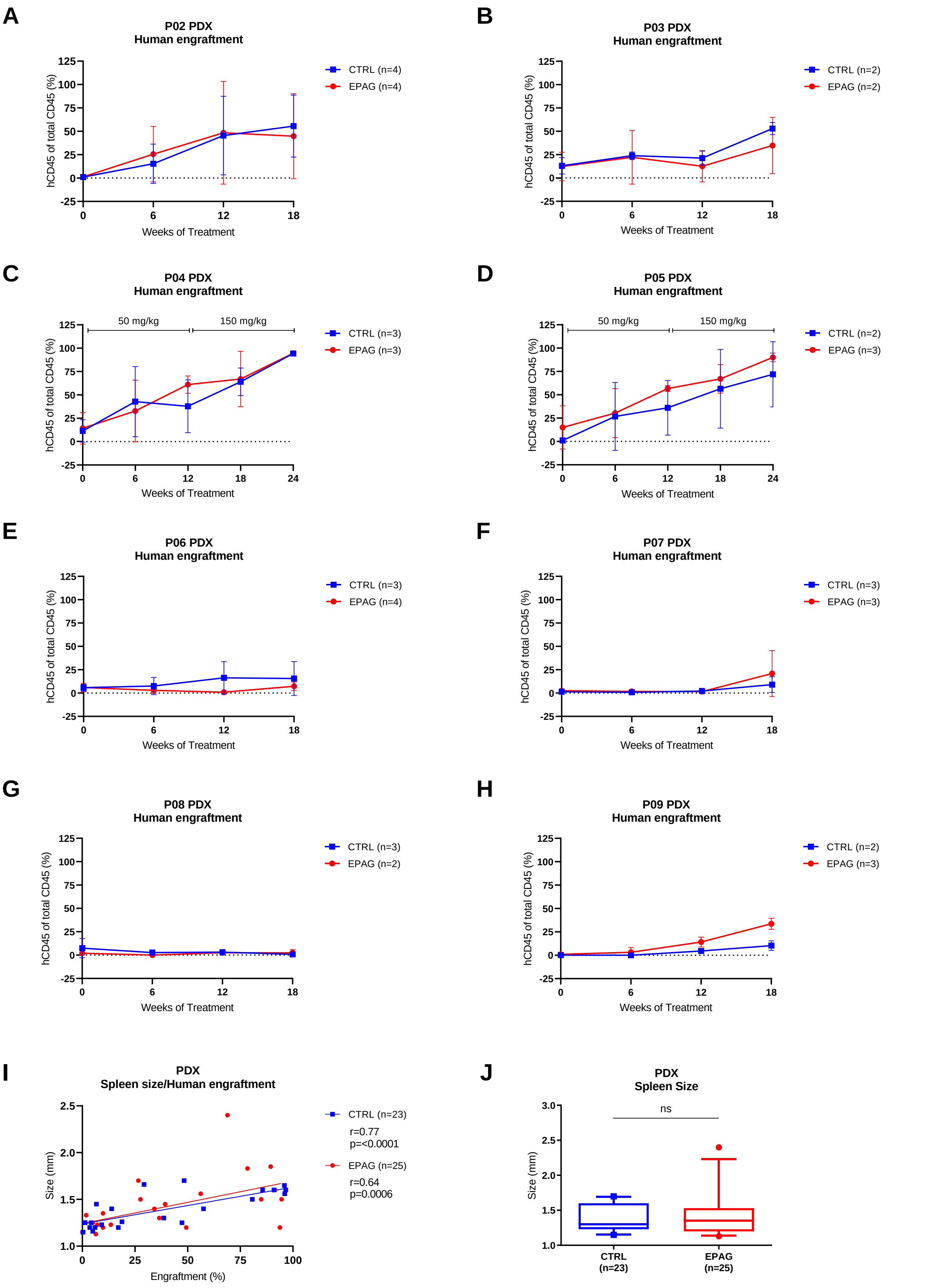

Supplementary Fig. S7 continued

K

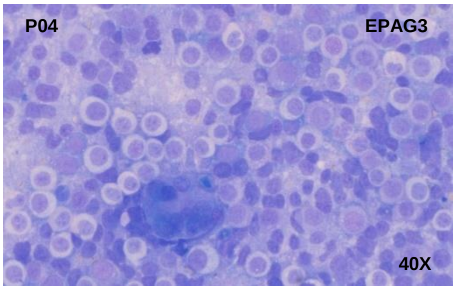

Supplementary Fig. S7 Engraftment in xenografts is not affected by EPAG treatment compared to vehicle control. (A-H) Human engraftment in the bone marrow (BM) for groups of eltrombopag- (EPAG, red) and vehicle-treated (CTRL, blue) xenografted mice of P02, P03, P04, P05, P06, P07, P08 and P09 in the course of 18 weeks of treatment. Engraftment was assessed every six weeks. Data was analyzed using unpaired, two-tailed t-test and is represented as means ± SD. **(I)** Correlation between engraftment and spleen size in xenografts of CTRL (r=0.77, p=<0.0001) and EPAG group (r=0.64, p=0.0006). Linear regression analysis was performed using Prism 8 (GraphPad Software) and Spearman's correlation coefficient was computed. Data is represented as means ± SD. ***p<0.001, ****p<0.0001; ns, not significant. **(J)** Spleen size of all xenografted mice from all patients for groups of n=25 EPAG and n=23 vehicle treatment. Data was analyzed using two-tailed Mann-Whitney U Test. Whiskers indicate 5-95% percentile. **(K)** BM smear of patient P04's xenograft EPAG3 at endpoint stained with May-Grünwald-Giemsa stain (magnification 40X). EPAG3 had to be sacrificed after 20 weeks due to excessive weight loss.

Supplementary Fig. S8

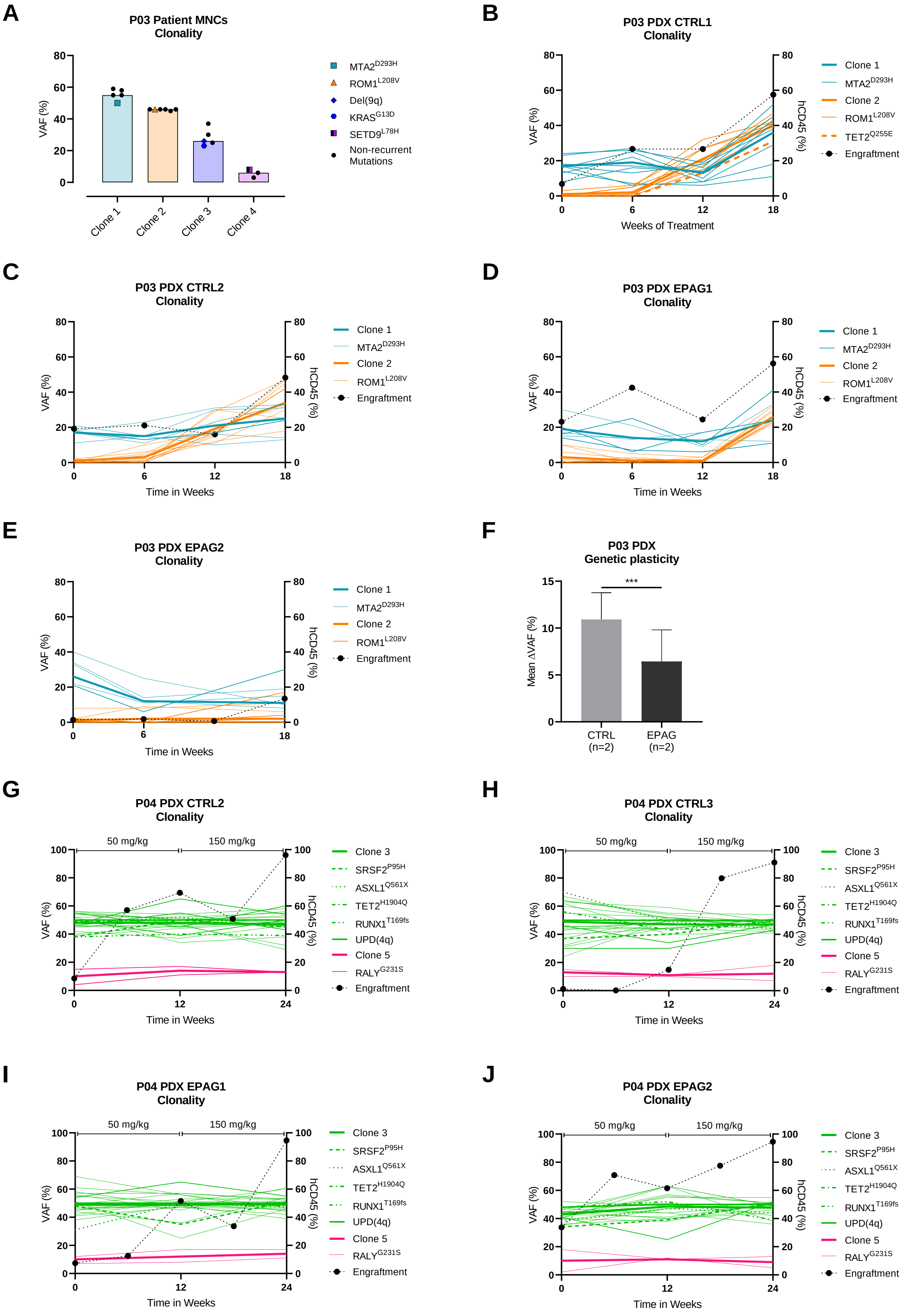

Supplementary Fig. S8 continued

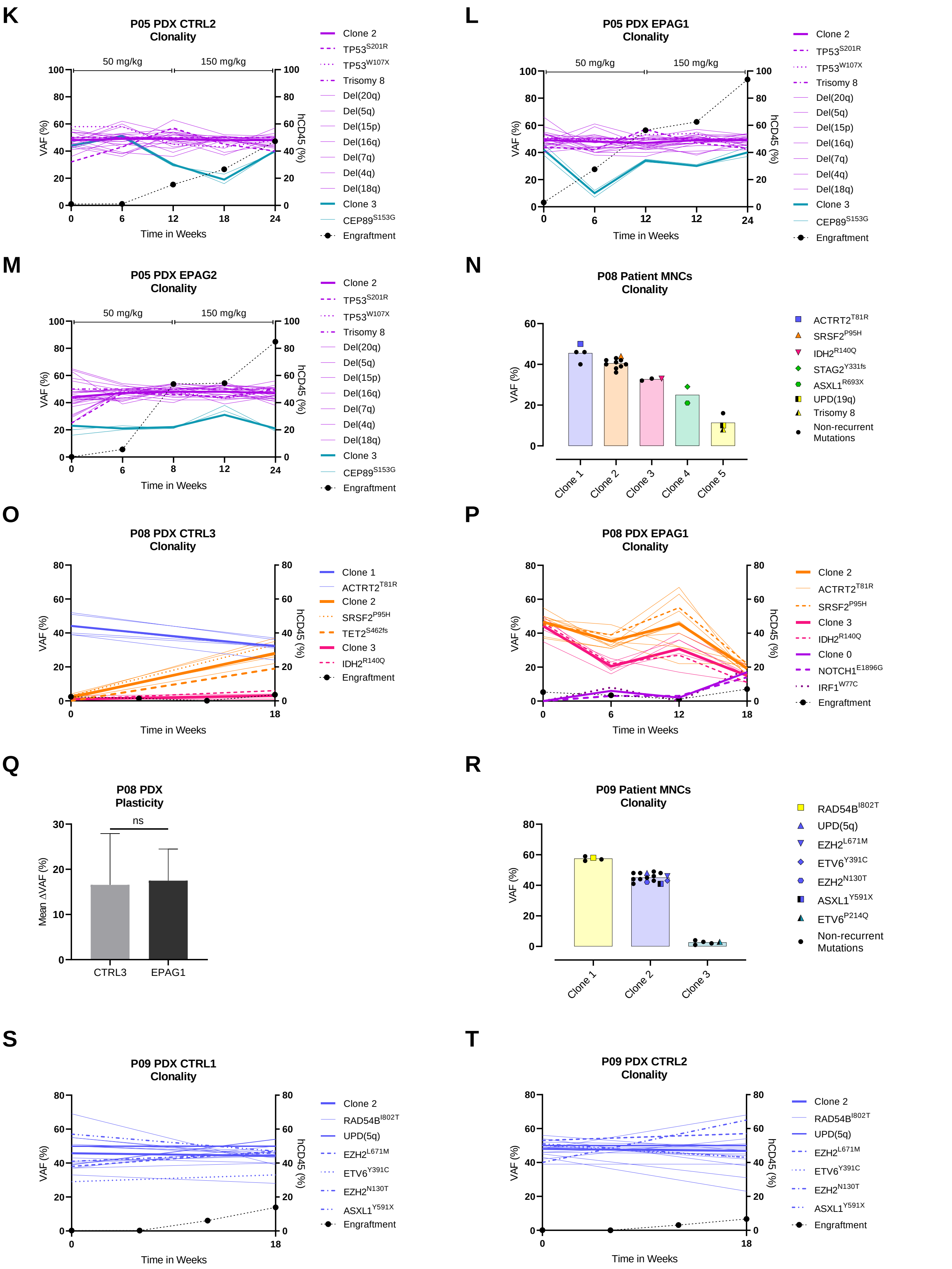

Supplementary Fig. S8 continued

U

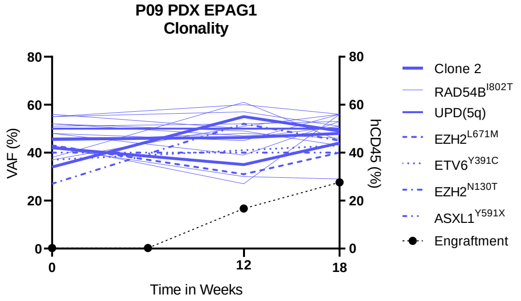

V

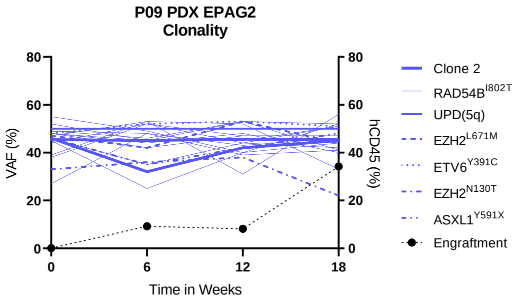

W

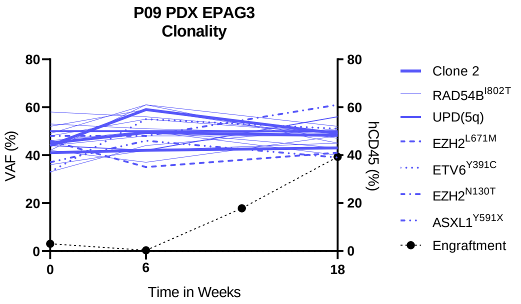

X

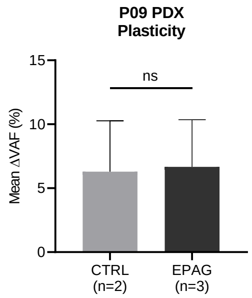

Y

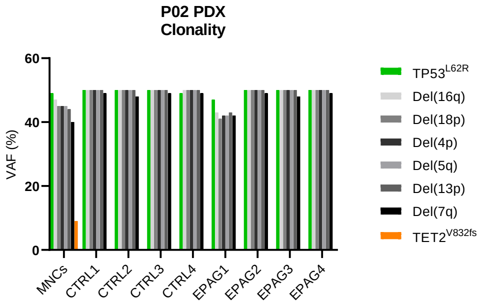

Z

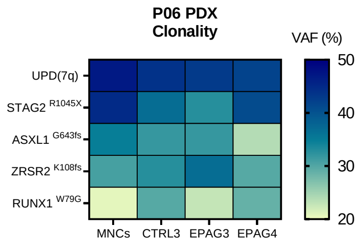

Supplementary Fig. S8 EPAG treatment does not promote increased clonal evolution compared to vehicle control. Clonal bone marrow composition of patients and their xenografts in the course of time reconstructed by clustering of variant allele frequencies (VAFs) using the bioinformatic tool SciClone. Mutations within the same cluster are equally colored. Thicker lines represent the mean value of the respective clone. Non-synonymous mutations are displayed with their superscripted respective amino acid change. Dotted black line describes course of engraftment in xenografts. **(A-E)** VAFs of patient P03's mononuclear cells (MNCs), and human CD45+ (hCD45+) cells from CTRL1 and EPAG1. **(F)** Total mean deltaVAF of P03's n=2 CTRL and n=2 EPAG mice. **(G-J)** VAFs of hCD45+ cells from patient P04's xenografts CTRL2+3 and EPAG1+2 throughout 24 weeks of experiment. Dose was escalated after 12 weeks from 50 mg/kg to 150 mg/kg for additional 12 weeks. **(K-M)** VAFs of hCD45+ cells from patient P05's xenografts CTRL2, EPAG1 and EPAG2 throughout 24 weeks of experiment. Dose was escalated after 12 weeks from 50 mg/kg to 150 mg/kg for additional 12 weeks. **(N-P)** VAFs of patient P08's MNCs, and hCD45+ cells from CTRL3 and EPAG1. **(Q)** Mean deltaVAF of CTRL3 and EPAG1. **(R-W)** VAFs of patient P09's MNCs, and hCD45+ cells from CTRL1+2 and EPAG 1-3 throughout 24 weeks of experiment. **(X)** Total mean deltaVAF of patient P09's xenografts CTRL1+2 and EPAG1-3 determined from all identified somatic mutations for any two consecutive WES time points. **(Y)** VAFs of patient P02's MNCs and sorted hCD45+ cells from its n=4 EPAG-treated (EPAG1-4) and vehicle-treated (CTRL1-4) xenografts at endpoint. **(Z)** Heatmap displaying VAFs of patient P06's MNCs, and hCD45+ cells from CTRL3 and EPAG3+4 at endpoint. Data in **F, Q** and **X** was analyzed using unpaired, two-tailed t-test and is represented as mean ± SD. ***p<0.001; ns, not significant. EPAG, eltrombopag.

Supplementary Fig. S9

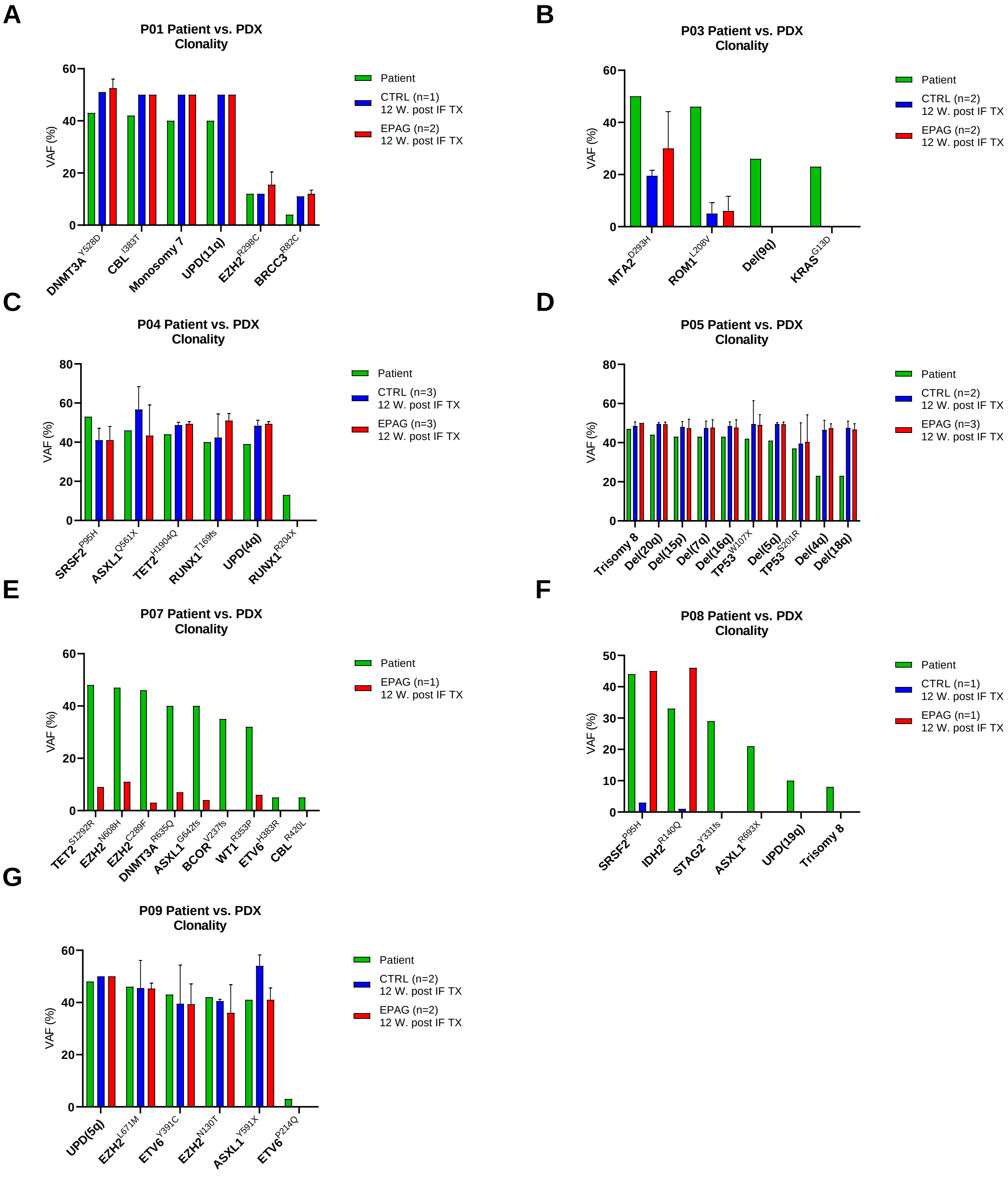

Supplementary Fig. S9 Primary patient samples and xenograft samples before treatment show similar mutational profiles. (A-G) Comparison of variant allele frequencies (VAFs) of MDS-specific mutations from primary patient samples of P01, P03, P04, P07, P08 and P09, and their respective patient-derived xenografts at 12 weeks post intrafemoral transplantation (IF TX) before start of treatment with either eltrombopag (EPAG) or vehicle (CTRL). All data is represented as means ± SD.
